# Typhoon- and pollution-driven enhancement of reactive bromine in the mid-latitude marine boundary layer

**DOI:** 10.1093/nsr/nwae074

**Published:** 2024-02-29

**Authors:** Shanshan Wang, Qinyi Li, Ruifeng Zhang, Anoop Sharad Mahajan, Swaleha Inamdar, Nuria Benavent, Sanbao Zhang, Ruibin Xue, Jian Zhu, Chenji Jin, Yan Zhang, Xiao Fu, Alba Badia, Rafael P Fernandez, Carlos A Cuevas, Tao Wang, Bin Zhou, Alfonso Saiz-Lopez

**Affiliations:** Shanghai Key Laboratory of Atmospheric Particle Pollution and Prevention (LAP^3^), Department of Environmental Science and Engineering, Fudan University, Shanghai 200438, China; Institute of Eco-Chongming (IEC), Shanghai 202162, China; Department of Atmospheric Chemistry and Climate, Institute of Physical Chemistry Blas Cabrera, CSIC, Madrid 28006, Spain; Department of Civil and Environmental Engineering, The Hong Kong Polytechnic University, Hong Kong 999077, China; Environment Research Institute, Shandong University, Qingdao 266237, China; Shanghai Key Laboratory of Atmospheric Particle Pollution and Prevention (LAP^3^), Department of Environmental Science and Engineering, Fudan University, Shanghai 200438, China; Centre for Climate Change Research, Indian Institute of Tropical Meteorology, Ministry of Earth Sciences, Pune 411008, India; Department of Chemistry, University of Colorado Boulder, Boulder, CO 80309, USA; Department of Atmospheric Chemistry and Climate, Institute of Physical Chemistry Blas Cabrera, CSIC, Madrid 28006, Spain; Shanghai Key Laboratory of Atmospheric Particle Pollution and Prevention (LAP^3^), Department of Environmental Science and Engineering, Fudan University, Shanghai 200438, China; Shanghai Key Laboratory of Atmospheric Particle Pollution and Prevention (LAP^3^), Department of Environmental Science and Engineering, Fudan University, Shanghai 200438, China; Shanghai Key Laboratory of Atmospheric Particle Pollution and Prevention (LAP^3^), Department of Environmental Science and Engineering, Fudan University, Shanghai 200438, China; Shanghai Key Laboratory of Atmospheric Particle Pollution and Prevention (LAP^3^), Department of Environmental Science and Engineering, Fudan University, Shanghai 200438, China; Shanghai Key Laboratory of Atmospheric Particle Pollution and Prevention (LAP^3^), Department of Environmental Science and Engineering, Fudan University, Shanghai 200438, China; Institute of Eco-Chongming (IEC), Shanghai 202162, China; Department of Civil and Environmental Engineering, The Hong Kong Polytechnic University, Hong Kong 999077, China; Institute of Environment and Ecology, Tsinghua Shenzhen International Graduate School, Tsinghua University, Shenzhen 518055, China; Sostenipra Research Group, Institute of Environmental Science and Technology (ICTA), Universitat Autònoma de Barcelona (UAB), Barcelona 08193, Spain; Institute for Interdisciplinary Science (ICB), National Research Council (CONICET), FCEN-UNCuyo, Mendoza M5502JMA, Argentina; Department of Atmospheric Chemistry and Climate, Institute of Physical Chemistry Blas Cabrera, CSIC, Madrid 28006, Spain; Department of Civil and Environmental Engineering, The Hong Kong Polytechnic University, Hong Kong 999077, China; Shanghai Key Laboratory of Atmospheric Particle Pollution and Prevention (LAP^3^), Department of Environmental Science and Engineering, Fudan University, Shanghai 200438, China; Institute of Eco-Chongming (IEC), Shanghai 202162, China; Department of Atmospheric Chemistry and Climate, Institute of Physical Chemistry Blas Cabrera, CSIC, Madrid 28006, Spain

**Keywords:** reactive bromine, atmospheric chemistry, marine boundary layer, atmospheric oxidation capacity, marine emission

## Abstract

Tropospheric reactive bromine is important for atmospheric chemistry, regional air pollution, and global climate. Previous studies have reported measurements of atmospheric reactive bromine species in different environments, and proposed their main sources, e.g. sea-salt aerosol (SSA), oceanic biogenic activity, polar snow/ice, and volcanoes. Typhoons and other strong cyclonic activities (e.g. hurricanes) induce abrupt changes in different earth system processes, causing widespread destructive effects. However, the role of typhoons in regulating reactive bromine abundance and sources remains unexplored. Here, we report field observations of bromine oxide (BrO), a critical indicator of reactive bromine, on the Huaniao Island (HNI) in the East China Sea in July 2018. We observed high levels of BrO below 500 m with a daytime average of 9.7 ± 4.2 pptv and a peak value of ∼26 pptv under the influence of a typhoon. Our field measurements, supported by model simulations, suggest that the typhoon-induced drastic increase in wind speed amplifies the emission of SSA, significantly enhancing the activation of reactive bromine from SSA debromination. We also detected enhanced BrO mixing ratios under high NO_x_ conditions (ppbv level) suggesting a potential pollution-induced mechanism of bromine release from SSA. Such elevated levels of atmospheric bromine noticeably increase ozone destruction by as much as ∼40% across the East China Sea. Considering the high frequency of cyclonic activity in the northern hemisphere, reactive bromine chemistry is expected to play a more important role than previously thought in affecting coastal air quality and atmospheric oxidation capacity. We suggest that models need to consider the hitherto overlooked typhoon- and pollution-mediated increase in reactive bromine levels when assessing the synergic effects of cyclonic activities on the earth system.

## Teaser

Typhoons can substantially elevate atmospheric bromine levels by enhancing sea-salt aerosol emissions, resulting in increased reactive bromine activation and ozone depletion. This underscores the previously underestimated impact of typhoon- and pollution-mediated reactive bromine chemistry on coastal air quality and atmospheric oxidation capacity.

## INTRODUCTION

Reactive bromine species are involved in many atmospheric chemical processes in both the troposphere and stratosphere. Reactive bromine species are known for altering atmospheric oxidation capacity via ozone (O_3_) destruction, HO_x_ and NO_x_ perturbations, as well as oxidation of sulfur species, volatile organic compounds (VOCs), and mercury [1,2]. On a global scale, the main sources of reactive bromine in the atmosphere are reported to be ocean-emitted bromocarbons and sea-salt aerosol (SSA) debromination [3,4]. On a regional scale, reactive bromine species (originating from blowing snow, SSA, brine-covered ice, frost flower and snowpack) initiate sharp tropospheric O_3_ depletion events in polar regions [5,6]. Given its importance, BrO is usually measured as the key species to represent the tropospheric reactive bromine. The detection of BrO over the remote ocean [7] and in volcanic plumes [8,9] led to wide interest in bromine chemistry. BrO mixing ratios exceeding 100 pptv over salt lake atmospheres (e.g. the Dead Sea) have been reported [10,11]. Anthropogenic emissions of gaseous inorganic bromine species and their significant impact on air quality were also recently proposed [12–14]. Bromine abundance and sources are uncertain in coastal areas due to limited observations and the influence of multiple natural and anthropogenic processes [15–21].

Polar cyclonic activity induces strong winds, resulting in larger amounts of blowing snow particles, facilitating autocatalytic chemical chain reactions of reactive bromine release from the particles (the so-called bromine explosion), causing polar surface O_3_ depletion events in the Arctic [22,23]. Cyclonic activities (also known as typhoons in the Northwest Pacific) substantially alter atmospheric composition over the ocean, coastal, and inland regions mostly through physical effects, e.g. atmospheric transport, precipitation, land-surface exchange, etc. [24–29]. However, the role of typhoons (and other extra-polar cyclones) in tropospheric reactive bromine sources and chemistry has not been reported.

Here, we report BrO observations using the Multi-Axis Differential Optical Absorption Spectroscopy (MAX-DOAS) technique on Huaniao Island (HNI) in the East China Sea (ECS) in the summer of 2018. Our observations, supported by model simulations, suggest that strong winds during typhoons amplify the emission of SSA, along with stronger pollution-induced activation of bromine from SSA, resulting in the unexpected enhancement of bromine species in the atmosphere, potentially leading to significant changes in O_3_ levels and atmospheric oxidation capacity in the region.

## RESULTS

### Observations of BrO

**Figure 1.**
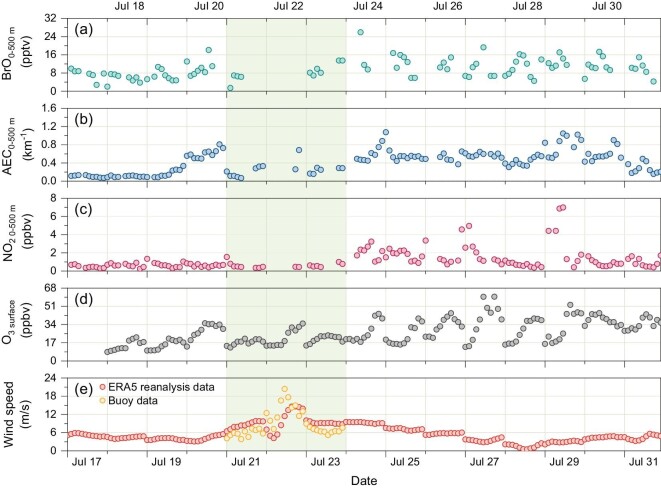
Time series of the daytime (a) BrO, (b) aerosol extinction coefficient, and (c) NO_2_ surface layer (0–500 m) concentration observed by MAX-DOAS at HNI site, China, (d) surface O_3_ observed by collocated SP-DOAS at HNI, as well as (e) wind speed from ERA 5 (https://cds.climate.copernicus.eu/) reanalysis data and measured by buoy (http://csdata.org/p/405/) at the location of HNI. The shaded area indicates the typhoon passing period. The missing data of MAX-DOAS retrieval ((a) to (c)) on July 22 is due to extreme weather conditions caused by typhoon activity.

Figure 1 presents the daytime (07 : 00–18 : 00 local time, LT) observed BrO volume mixing ratios (VMR) averaged from the surface up to 500 m, other trace gases and aerosol extinction coefficient (AEC) together with the surface O_3_ concentrations. We observed consistently high levels of BrO with a daytime average of ∼9.7 ± 4.2 pptv in marine boundary layer (MBL) during a 15-day field campaign on HNI, with a distinct enhancement by the typhoon activity (Text S1). In polar regions [6] and salt lakes [10,11,30], BrO mixing ratios exceeding 10 pptv were commonly reported. However, in the MBL, previous studies (summarized in Table S1; Text S2) reported that: in clean MBL, daytime mean maxima mixing ratios were typically around 2–3 pptv, with peaks reaching 6 pptv [7,31–35]; up to 7.5 ± 1.0 pptv was reported in one study in a semi-polluted coastal environment [15]; ∼10 pptv was observed only once during a cruise-based study along the west African coast [17]. Compared to the previous reports, our study consistently measured elevated BrO (∼10 pptv) in the MBL.

The observed columnar integrated aerosol optical depth (AOD), BrO, and NO_2_ vertical column densities (VCDs) below 2 km were averaged at 0.53, 3.02 × 10^13^ molec. cm^−2^, and 2.34 × 10^15^ molec. cm^−2^, which is much higher than those over remote ocean areas. These higher values are due to the short distance to the continent with noticeable impacts of the anthropogenic emissions of air pollutants [36–39]. The vertically resolved AEC, BrO, and NO_2_ presented a declining trend with the increase in height (Fig. S2). Aerosol and BrO spread from the sea surface up to 1 km, whereas NO_2_ is mainly concentrated in the lowest layer (up to 0.4 km). This dissimilar vertical distribution may be explained by their different sources and transport in the MBL. In addition, the day-to-day diurnal patterns of the trace gases and aerosols changed as typhoon ‘Ampil’ developed and passed through the measurement site.

### Contribution of natural and anthropogenic sources

**Figure 2.**
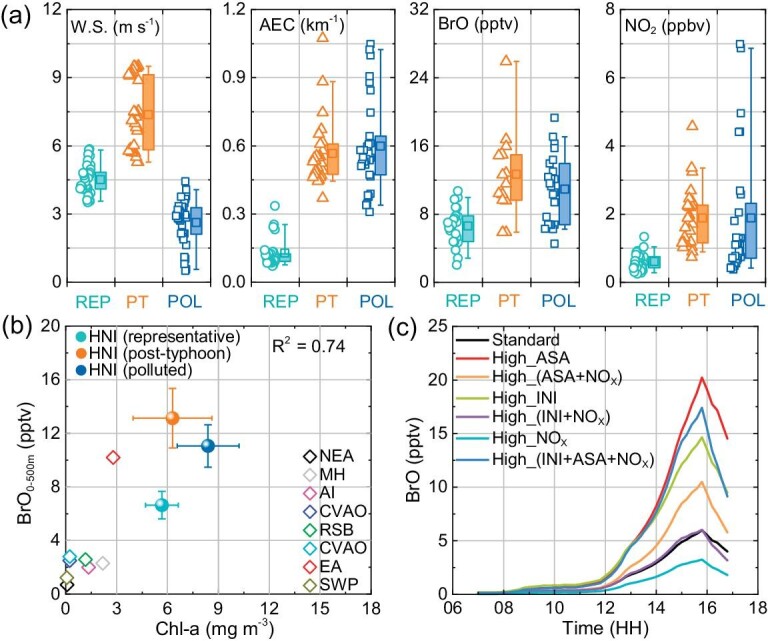
Relationship between BrO VMR with (a) wind speed, AEC, and NO_2_ and (b) Chl-a, on different characterized periods of representative (REP; July 17 to 19), post-typhoon (PT; July 24 to 26) and polluted days (POL; July 27 to 29). The bottom and top edges of the box in (a) indicate the range of values between the first and third quartiles (the 25th and 75th percentiles). The marker inside the box indicates the mean value. The whiskers indicate the range of 5th and 95th percentiles. Diamonds in (b) indicate the previous BrO measurements (summarized in Table S1) and balls in (b) are the averages for three characterized periods with an error bar of standard deviation. (c) THAMO model simulation of daytime variation of BrO in response to various parameters, aerosol surface area density (ASA), initial bromine levels (INI), ambient NO_x_ levels (NO_x_), and the combination of them (Table S2).

Figure 2a shows a comparison of the wind speed, AEC, BrO and NO_2_ mixing ratios at the surface layer (0–500 m) for different periods, characterized as representative MBL conditions (REP; July 17 to 19), post-typhoon (PT; July 24 to 26) and polluted conditions (POL; July 27 to 29). During the representative MBL conditions with moderate wind speeds and lower NO_2_ and aerosol, the BrO levels (∼6 pptv) are comparable to observations at Cape Verde (with a mean maximum value of 5.6 ± 1.0 pptv) [7]. During the post-typhoon period, significant increases in AEC suggested higher SSA concentrations and stronger SSA debromination, which resulted in higher BrO mixing ratios. The emission of SSA into the atmosphere takes place through air bubbles bursting at the ocean surface and is positively and exponentially correlated with wind speed [40,41]. The observed wind direction and the air mass back-trajectories confirmed that the air masses predominantly came from the open ocean during the measurement period (Fig. S3). It is noteworthy that higher NO_2_ levels (>4 ppbv) were observed during the last few days of the campaign, during which the BrO mixing ratios sustained at relatively high levels of ∼10 pptv.

Chlorophyll a (Chl-a) is an important indicator of algal biomass in aquatic ecosystems, which can be used as a proxy to provide an estimate of phytoplankton biomass in seawater [42]. Bromocarbons, e.g. bromoform (CHBr_3_), dibromomethane (CH_2_Br_2_), dibromochloromethane (CHBr_2_Cl), and bromodichloromethane (CHBrCl_2_), are ubiquitous in the oceans, and are mainly formed directly or indirectly by macro- and microalgae [43–46]. Consequently, enhanced Chl-a concentration implies higher algal biomass and stronger release of oceanic bromocarbons [47]. As per the previous results (Table S1), the relation between BrO VMR and Chl-a concentrations derived from satellite observations, indicate organic sources of bromine species (Fig. 2b). The measured data agree with the response of oceanic chlorophyll-a abundances (*R*^2^ = 0.74). A previous study reported high BrO mixing ratios (up to 10.2 ± 3.7 pptv) in the East Atlantic (‘EA’ in Fig. 2b) during a cruise passing through the Mauritanian upwelling system suggesting a potentially important role of oceanic biogenic sources for bromine species [48,49]. Cyclone-driven strong disturbance, mixing, and upwelling in the upper layer of the oceans can enhance the coastal euphotic layer, which is reported to induce the phytoplankton and algal bloom [50,51]. Chl-a abundances in ECS are significantly higher than in other marine regions, possibly related to the eutrophication in the coastal seas of China [52–54]. The additional segmented observation at HNI in the spring of 2018 also suggested the even higher Chl-a and comparable levels of BrO to the representative periods in summer (Fig. S4). The complex linkage between algal bloom emission and its feedback on atmospheric and fluvial nutrients highlights the importance of investigating the biogeochemical cycling of reactive bromine species [55,56].

Figure 2c demonstrates the response of the BrO levels to various factors as simulated by a one-dimensional chemical and transport model (THAMO) [57,58] constrained with available observations at HNI (Text S5; Table S2). The results show that higher NO_x_ levels lead to lower BrO, higher initial inorganic bromine levels lead to higher BrO, and higher aerosol surface areas lead to enhanced aerosol release and recycling of inorganic bromine resulting in higher BrO. The combination of higher NO_x_ and higher initial inorganic bromine species (or higher SSA) leads to similar levels of BrO, suggesting the possibility of high BrO under high NO_x_ environments with the presence of sufficient bromine precursors. Note that high BrO levels have been previously reported in high NO_x_ environments [15]. Recent observations at a polluted coastal site in Hong Kong reported significant daytime levels of molecular bromine, revealing a potentially large bromine source through nitrate aerosol photolysis [21]. Here we highlight the competing role of NO_x_ in controlling the level of BrO: (1) NO_2_ reacts with BrO, directly reducing BrO mixing ratio and (2) NO_2_ forms nitrate aerosol which further photolyzes (with the presence of bromide-containing aerosol) to produce gaseous reactive bromine, therefore indirectly enhancing BrO level. The simulated responses of BrO to these key factors are in line with the observation-derived relationship (Fig. 2a).

In addition to the chemical activation mechanism emphasized above, acid displacement and gas-particle processes are also affected by the typhoon, influencing the source as well as the sink of reactive bromine gases: (1) a larger amount of SSA and more fresh SSA implies more Br^−^ leading to more HBr being transferred to the gas phase, under favourable conditions like similar or higher temperature; (2) the strong winds, induced by typhoons, uplift more sea spray drops, resulting in more liquid water in the atmosphere and larger aerosol surface area, leading to more uptake of gaseous bromine onto the liquid drops, hence a sink for reactive bromine gases. We note that the heterogeneous uptake of HOI on SSA producing IBr is also considered in our study [58], and contributes to the total bromine. Our modelling results (HAL case) show that the simulated IBr mixing ratio is only 0.24 pptv at HNI during the observation period (July 16 to 31); the sum of species (i.e. HOBr, BrNO_2_, BrNO_3_, Br_2_, and BrCl) related to the bromine self-activation processes (Table S3), however, is ∼50 times larger (14.0 pptv), therefore, dominating the source of bromine in this region.

We have also considered the influence of oceanic dimethyl sulfide (DMS) on BrO levels. We have conducted a sensitivity case (wthDMS) including the oceanic DMS sources using the method developed by Li *et al.* [59], and the DMS oxidation by halogen radicals (BrO, Cl, and IO) following Veres *et al.* [60]. Our sensitivity test suggests that the inclusion of DMS source and chemistry reduces the simulated BrO at HNI during the observation period (July 16–31) by ∼10% (Fig. S5), which is non-negligible as shown in previous works (e.g. Hoffmann *et al.* [61]). However, we note that the temporal pattern of simulated BrO mixing ratio before and after the typhoon (Fig. S5), i.e. typhoon-induced bromine enhancement, remains the same with the inclusion of DMS source and chemistry.

### Impact of bromine on O_3_

**Figure 3.**
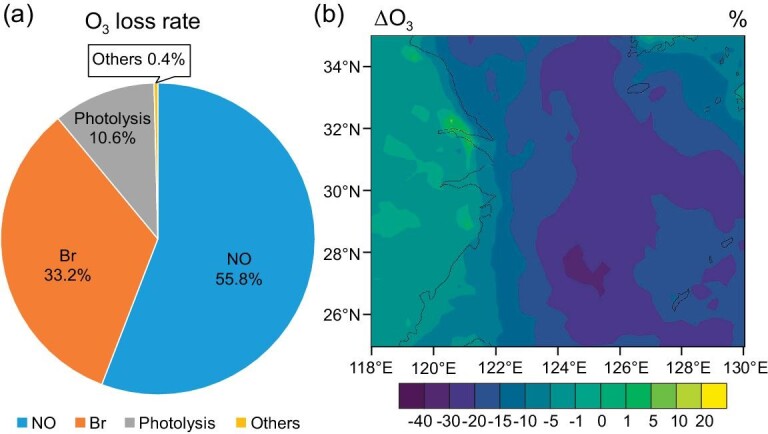
Bromine chemistry effects on O_3_ during July 17–31, 2018. (a) THAMO-simulated contribution of individual channels to O_3_ loss rate at HNI. The O_3_ loss rate of ‘Others’ mainly includes O_3_ reactions with OH, HO_2_, and NO_2_. The loss of O_3_ due to chlorine and iodine processes are not included in the THAMO calculation. (b) WRF-Chem simulated spatial distribution of the bromine effects (between noBr and HAL cases in percentage) on the O_3_ mixing ratio at the surface layer (the first modelling layer, from ground/sea level to ∼40 m). Chlorine and iodine sources and chemistry are included in both the noBr and HAL WRF-Chem cases.

Such elevated levels of BrO imply a significant role of bromine on atmospheric ozone in this region during the typhoon period. One-dimensional model (THAMO) simulations constrained with observations at HNI (Text S5; Table S2) indicate that Br chemistry (33.2%) is the second largest contributor to the total O_3_ destruction (Fig. 3a), after NO (55.8%). Photolysis of O_3_ makes a smaller fraction of 10.6% and other minor loss pathways contribute 0.4%, including O_3_ reactions with OH, HO_2_, and NO_2_. By neglecting reactive bromine chemistry, one might leave out a critical fraction of the O_3_ loss in this region (clean open ocean to semi-polluted coast), influencing the representation of background air in simulating air quality in the Yangtze River Delta (YRD) region. Please note that by using the observation-based model, we only quantify the bromine impacts on O_3_ loss but we are not able to estimate the bromine influence on O_3_ production or O_3_ concentration, due to the lack of concurrent observations of volatile organic compounds (VOCs).

To further explore the potential impacts of typhoon-driven reactive bromine on O_3_ abundance in this region, we apply an emission-based chemical transport model (Weather Research and Forecasting model coupled with Chemistry, WRF-Chem; Text S6; Table S3). WRF-Chem simulation results (HAL case with the complete halogen species sources and chemistry) reproduce the observed temporal trend in the BrO mixing ratio with a noticeable increase under the influence of the typhoon (Fig. S5). WRF-Chem results (Fig. 3b) show that bromine reduces the O_3_ mixing ratio (R10 in Supplementary material) in most of the oceanic regions by as much as ∼40%, and increases O_3_ in the continental area (up to ∼10%; R3 to R8 in Supplementary material), where large anthropogenic emissions of NO_x_ and VOCs are located. Such an enhancement in O_3_ abundance due to bromine chemistry is comparable to the deterioration of ozone pollution in the last 10 years in eastern China [62,63]. Omitting such effects of reactive bromine chemistry leads to potential uncertainty in formulating O_3_ pollution control strategies. The cleansing effect of bromine chemistry on tropospheric O_3_ has been widely documented [1,2]; the enhancement effect of bromine on O_3_ and secondary aerosol has also been reported in northern China [14]. Therefore, the competing role of bromine chemistry in regulating oxidation capacity and air quality highlights the need to consider the typhoon-driven enhancement of bromine levels in typhoon impact assessments in the East China Sea region.

## DISCUSSION

Cyclones, characterized by an intense low-pressure system, are one major atmospheric event affecting atmospheric compositions and dynamics [64,65]. Cyclones significantly impact the summertime air quality in the mid-latitude regions, e.g. East Asia [26,66–68], South Asia [69,70], and Central and Northern America [71,72]. By combining multiple tools, including direct field measurement, meteorological data, satellite observations, measurement-based box modelling, and emission-based regional modelling, the present work establishes a link from cyclone activity, to the substantially increased surface wind speeds, to the amplified SSA emission, to the larger fresh SSA abundance in the MBL, and eventually to the enhanced bromine release. Such a bromine release, similar to the polar bromine explosion events [73–75], is a self-accelerated process, which is terminated when SSA is deposited to the sea surface (resulting in an atmospheric lifetime up to a few days [76]) or depleted in bromide content. Such interaction between two natural processes (cyclone activity and SSA heterogeneous process) is expected to occur in all parts of the ocean with strong cyclone activities, e.g. West Pacific, Indian Ocean, and the Atlantic Ocean, and further affect the atmospheric composition and processes over coastal areas in tropical and subtropical regions. Under the changing climate, warming of the ocean surface is likely fuelling and increasing the intensity of tropical cyclones [77], which might lead to increased cyclone-driven bromine release. The current study focuses on the enhancement in reactive bromine abundance in the MBL due to typhoons, while it is possible that the sources of other reactive halogens (e.g. chlorine and iodine) and non-halogen biologically produced emissions (i.e. DMS) are also altered during typhoons. The reader is referred to Text S8 for a more detailed discussion on such a possibility.

The clear positive correlation between the field-observed BrO and satellite-derived Chl-a (Fig. 2b) suggests that either BrO depends on Chl-a (through biogenic organic bromine emission from the ocean), or both BrO and Chl-a depend on the same variable/process. During the representative days (Jul 17–19), applying a lower emission flux of oceanic organic bromine in WRF-Chem (lowORG case) leads to a noticeable reduction (∼20%) in the simulated BrO compared to the HAL scenario (Fig. S5); therefore, in terms of long-term effects, a noticeable amount of atmospheric reactive bromine originates from oceanic organic bromine emission (indicated by the Chl-a). During the days of typhoon passing (July 21–23), the simulated BrO in lowORG and HAL only shows a small difference (<5%; Fig. S5); hence, the oceanic biogenic process (Chl-a) has little effect in the short term on atmospheric reactive bromine considering the longer lifetime (≥14 days) of ocean emitted organic bromines [78,79]; instead, we propose that during the typhoon passing period, Chl-a and SSA are influenced by the same process, e.g. air-sea exchange. This cyclone-driven process and impact were pointed out by a simulation study in which a hypothetical cyclone caused an extremely large release of CHBr_3_ from the macroalgae farm into the atmosphere, resulting in significant CHBr_3_ mixing ratios in the atmosphere, especially at altitudes below 5 km [80].

**Figure 4.**
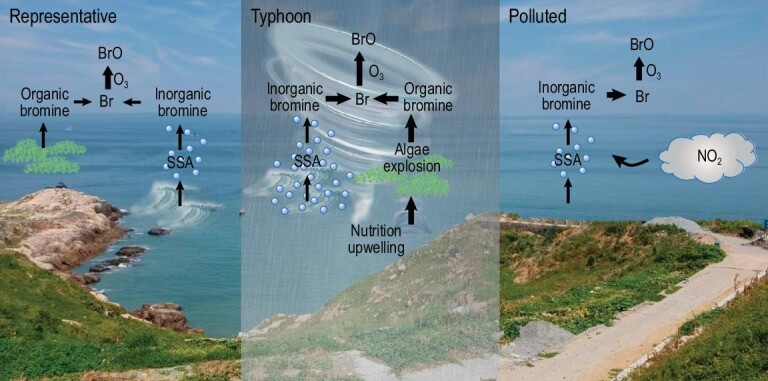
Schematic figure of the reactive bromine source and chemistry during the representative (left), typhoon (middle) and polluted (right) periods. The typhoon activities induce larger wind speeds over the ocean, enhancing the emissions of SSA, leading to larger sources of reactive bromine, which, in the presence of O_3_, generates more BrO, i.e. ‘typhoon→larger wind speed→more SSA→more bromine’. The polluted condition with a high abundance of both NO_2_ and initial inorganic bromine species (or higher SSA) can sustain a similar enhancement of BrO, suggesting the possibility of high BrO under high NO_x_ environments with the presence of sufficient bromine precursors. The background picture shows the HNI coast in which the observation was conducted.

Typhoon activity amplifies the air-sea exchange (‘Typhoon’ in Fig. 4) which (1) facilitates the mixing of nutrients in the seawater causing macroalgae blooms, and (2) breaks the sea-water bubbles and forms more SSA, resulting in higher BrO mixing ratios in the MBL. As a two-way process, air-sea exchange, significantly enhanced by the typhoon activity, may also lead to an enhanced sink of halogens, which needs further investigation. Despite the well-established chain of evidence suggesting the enhanced bromine sources from SSA-dehalogenation, which was impacted by the large windspeeds resulting from the typhoon activity, we note that further measurement and laboratory studies oriented to investigate the complex processes during typhoons should be performed in order to refine the proposed mechanism.

One interesting phenomenon is the co-existence of high BrO and high NO_2_ (‘Polluted’ in Fig. 4). A previous work [15] suggested that such co-existence could only occur if an unknown local source of bromine (or rapid recycling processes) existed. In our THAMO model simulations, we show that a larger initial level of reactive bromine could cancel out the effect of a higher NO_x_ level and maintain a similar simulated BrO mixing ratio. The WRF-Chem results (noANT and HAL cases; Fig. S5), however, show that during the simulation period, the currently known sources of anthropogenic bromine emission [14] have little effect on the simulated BrO level at HNI, suggesting that there could be a previously unrecognized source of reactive bromine that supports the elevated level of BrO during high NO_x_ days. A possible source is the nitrate-initiated bromine release from bromide-containing aerosols, e.g. SSA [21]. Future work is needed to further quantify the competing role of NO_x_ in bromine source and chemistry.

While the physical/meteorological factors (wind and precipitation) are known to affect O_3_ during typhoons [81–83], here we show that reactive bromine chemistry, enhanced by typhoon activity, could also lead to a significant change in surface O_3_ in coastal areas. Such a chemical effect of typhoons on the coastal air quality, not reported until now, is worthy of further investigation. The continental outflow with elevated levels of NO_x_ and nitrate aerosol might lead to enhanced bromine activation from SSA and higher bromine burden in the coastal air and result in a stronger chemical effect on coastal O_3_ pollution.

## METHODS

Detailed descriptions of all methods and materials are presented in the Supplementary material. Briefly, the observation of BrO, NO_2_ and aerosol were performed by MAX-DOAS instrument in the field measurements in July 2018 at the Huaniao Island site in the MBL of ECS [39] (Text S3). The spectral analysis and profile retrieval are descried in Text S4. The Tropospheric Halogen Chemistry Model (THAMO [15,58]) is used to show the response of BrO levels to a few critical factors (Text S5), while the regional chemical transport model (WRF-Chem), incorporated with comprehensive bromine chemistry, is utilized to investigate the potential influences of various sources on the abundance and impacts of reactive bromine species at HNI and the surrounding region (West Pacific and East Asia) (Text S6). Designs of the simulations and input settings are presented in Tables S2, S3 and S5.

## Supplementary Material

nwae074_Supplemental_File
