# Supplementary material for: Typhoon- and pollution-driven enhancement of reactive bromine in the mid-latitude marine boundary layer
Source: Natl Sci Rev. 2024 Feb 29;11(4):nwae074. doi: 10.1093/nsr/nwae074 (PMC11018124; doi:10.1093/nsr/nwae074)
Supplement: nwae074_Supplemental_File [file nwae074_supplemental_file.pdf]

Supplementary Information for

Typhoon- and pollution-driven enhancement of reactive bromine in the mid-latitude marine boundary layer

Shanshan Wang[1,2,#], Qinyi Li[3,4,5,#], Ruifeng Zhang[1], Anoop Sharad Mahajan[6], Swaleha Inamdar[7], Nuria Benavent[3], Sanbao Zhang[1], Ruibin Xue[1], Jian Zhu[1], Chenji Jin[1], Yan Zhang[1,2], Xiao Fu[4,8], Alba Badia[9], Rafael P. Fernandez[10], Carlos A. Cuevas[3], Tao Wang[4], Bin Zhou[1,2,*], Alfonso Saiz-Lopez[3,*]

[1] Shanghai Key Laboratory of Atmospheric Particle Pollution and Prevention (LAP[3]), Department of Environmental Science and Engineering, Fudan University, Shanghai, 200438, China

[2] Institute of Eco-Chongming (IEC), Shanghai 202162, China

[3] Department of Atmospheric Chemistry and Climate, Institute of Physical Chemistry Blas Cabrera, CSIC, Madrid 28006, Spain

[4] Department of Civil and Environmental Engineering, The Hong Kong Polytechnic University, Hong Kong 999077, China

[5] Environment Research Institute, Shandong University, Qingdao 266237, China

[6] Centre for Climate Change Research, Indian Institute of Tropical Meteorology, Ministry of Earth Sciences, Pune, 411008, India

[7] Department of Chemistry, University of Colorado Boulder, Boulder, CO 80309, USA

[8] Sostenipra Research Group, Institute of Environment and Ecology, Tsinghua Shenzhen International Graduate School, Tsinghua University, Shenzhen 518055, China

[9] Institute of Environmental Science and Technology (ICTA), Universitat Autònoma de Barcelona (UAB), Barcelona 08193, Spain

[10] Institute for Interdisciplinary Science (ICB), National Research Council (CONICET), FCEN-UNCuyo, Mendoza M5502JMA, Argentina

# These authors contributed equally.

* Corresponding author: Alfonso Saiz-Lopez (a.saiz@csic.es); Bin Zhou (binzhou@fudan.edu.cn).

Contents of this file

Text S1 to S10

Figures S1 to S10

Table S1 to S5

Text S1. Field measurements

Field measurements were performed from July 17 to 31, 2018, at an MBL site of Huaniao Island site (HNI, 30.85° N, 122.69° E, ~60 m a.s.l.) located in the ECS, approximately 70 km away from the nearest shore (Fig. S1) (Zhang et al., 2022). Due to the absence of industrial and traffic activities, the HNI site is an ideal background MBL environment (Li et al., 2016). Due to the typical monsoon characteristic, the anthropogenic pollutants from adjacent regions and distant inlands can be transported to the coastal areas, and even further reach the surrounding marginal seas, i.e., ECS (Nakamura et al., 2005; Pani et al., 2017). So HNI site is also reported to be a receptor of transport of continental outflow on the MBL of ECS (Guo et al., 2019; Yang et al., 2020; Wang et al., 2019).

Due to the prevailing summer monsoon climate, the south to the southeast wind is dominant at the HNI site during this campaign, as the wind rose shown in Fig. S3. Also, the dependency of NO_2 VMR at the surface layer observed by MAX-DOAS on wind conditions during the daytime between 07:00 and 18:00 (LT) together with the back-trajectories of the air masses was displayed, which suggested the weak influence of continental outflow during the campaign. The typhoon "Ampil" occurred in the ECS, eventually passed along HNI and made landfall on mainland China during the campaign. More details about the typhoon are presented in "Text S10. Typhoon 'Ampil'" following.

Text S2. Previous field measurements of BrO in MBL

As a key indicator of the reactive bromine chemistry in the troposphere, BrO has been detected over polar regions, salt lakes and volcanic plumes for its strong sources. In MBL, previous measurements of BrO were carried out in the two mid-latitude coastal locations, Mace Head (Saiz-Lopez et al., 2004) and the Canary Islands (Leser et al., 2003), and also at the equatorial mid-ocean site of Cape Verde (Read et al., 2008; Mahajan et al., 2010), etc., most of them were located in the Atlantic Ocean area. So far, the mid-latitude MBL of the Pacific Ocean area is still a vacancy for measurements.

As summarized in Table S1, BrO shows the typical daytime mean maxima around 2~3 pptv in the clean MBL (Laser et al., 2003; Saiz-Lopez et al., 2004; Keene et al., 2007; Read et al., 2008; Mahajan et al., 2010; Le Breton et al., 2017), and higher BrO was reported in a polluted coastal environment when NO_2 levels exceeded 1 ppb (Mahajan et al., 2009). An extremely high BrO exceeding 10 pptv was observed during the ship cruise along the west African coast area, which might be due to the halogenated hydrocarbons emitted by tropical halophytes growing in Mauritania and organohalogens emitted from algae (Martin et al., 2009). Here, it is the first time to report the BrO abundance in MBL of mid-latitude in East Asia. Compared with previous MBL measurements, we found significantly higher daytime BrO (~9.0 pptv) than previous measurements under semi-polluted or polluted environments.

In addition to the reported BrO dataset in the main text, we have conducted short-term and segmented observations in the spring of 2018 to test the instrument performance. As shown in Fig. S4, the spring observation data are also categorized into three conditions: (1) middle wind speed (3-6 m/s), (2) high wind speed (> 6 m/s), and (3) lower wind speed (≤3 m/s) combined with higher NO_2 levels (> 2 ppbv), which corresponds to representative, post-typhoon, and polluted days, respectively, in the original manuscript. These additional results also show similar conclusions as highlighted in our manuscript: (1) the Chl-a in this region in spring 2018 is larger than those reported previously in other regions, and the representative mixing ratios of BrO in Spring 2018 are ~5 pptv, which is similar to those in July 2018; (2) larger wind speeds and SSA (AEC) lead to higher BrO levels, even when the NO_2 levels are similar to the representative days; and (3) higher NO_2 levels also contribute to the increase of BrO compared to the representative days.

Text S3. MAX-DOAS and short-path DOAS instrument

Due to the sensitivity to the aerosol and trace gases abundances in the lower troposphere (Platt and Stutz, 2008), the MAX-DOAS instrument was used to observe the profiles of aerosol, BrO, and other trace gases. Briefly, it consists of a receiving telescope, a spectrometer and a computer to operate the system (Zhang et al., 2018; Cheng et al., 2019; Zhang et al., 2022). The telescope collects the scattered sunlight at different lower elevation angles (i.e., 0°, 1°, 2°, 3°, 5°, 7°, 10°, 30°) and the zenith direction (90°) sequentially driven by the stepper motor, which takes about 12 min for each cycle. The received scattered sunlight was converged by the lens with a focal length of 101.6 mm onto the fibre bundle connected to the spectrometer. Afterwards, the light signal was detected by the charge-coupled device (CCD) and recorded by the spectrometer (Ocean Optics, QE65 Pro) with a wavelength range from 290 to 473 nm and a resolution of about 0.5 nm full width half maximum (FWHM). To avoid the interference of direct sunlight, the telescope was pointed to the azimuth angle of 330° from the north clockwise. The signal of the dark current was extracted automatically from background measurements taken each night.

In addition, an active DOAS with a short light path was collocated to measure surface O_3 concentration (~35 m a.s.l.). The so-called short-path DOAS (SP-DOAS) consists of two telescopes with a diameter of 210 mm as transmitter and receiver, respectively, a 35 W Deuterium lamp as light source and a spectrograph with a range of 195-455 nm (Wang et al., 2015; Guo et al., 2021). The light path between the transmitter and the receiver is 180 m. The exposure time of each scan was adjusted automatically according to the light intensity and the average temporal resolution of the system was about 1 min.

Text S4. Spectral analysis and profile retrieval

The DOAS algorithm is based on the Beer-Lambert law, which describes the extinction of radiation through the atmosphere (Platt and Stutz, 2008). For MAX-DOAS measurement, the spectral analysis generates the measured SCD (slant column density), defined as the integral of the trace gas concentration along the entire optical path including the SCDs in the troposphere and the stratosphere. To eliminate the stratospheric contribution to the SCD, the zenith spectrum was adopted as the Fraunhofer reference spectrum (FRS) for the measured spectra of lower elevation angles during each measurement scanning cycle, assuming the stratospheric absorption would be at the same level within a cycle. Consequently, the SCD of the trace gas measured at each lower elevation angles is represented by the DSCD (differential SCD), which is defined as the light-path integrated trace gas concentration relative to a reference spectrum (Wagner et al., 2011).

Before spectral analysis, we used the ratio of light intensity at 330 nm to that at 390 nm served as colour index (CI) to filter the cloud conditions (Wagner et al., 2016). The QDOAS software (http://uv-vis.aeronomie.be/software/QDOAS/) was applied to analyze the measured spectra. Besides the absorption of interference trace gases, a synthetic ring spectrum (calculated by QDOAS) was included in the spectral fitting analysis. Table S4 summarizes the relevant configurations for the spectral analysis of O_4 (oxygen dimer O_2–O_2), BrO and NO_2.

Figure S7 shows the example of spectral fitting for O_4, BrO and NO_2, which displays good performance with obvious absorption structures and low residuals. Before the profile retrieval, the threshold of RMS $<1.0 \times 10^{-3}$ and the ratios of DSCD errors to DSCDs of 10% for O_4 and NO_2, and 50% for BrO were applied for the DSCDs to filter the unreliable data. The proportion of O_4, BrO and NO_2 passing the filter criteria accounted for 82.3%, 50.5% and 77.6% of the total daytime data, respectively. Due to the bad weather caused by typhoon Ampil, considerable spectral fitting results during typhoon interference days, i.e., 20 to 23 July, did not pass the filter.

Combined with the forward radiative transfer model, the measured DSCDs data set can be further retrieved to obtain the vertical profile and vertical column density (Platt and Stutz, 2008). Based on the optimal estimation method (OEM), the vertical profiles of aerosol extinction and volume mixing ratios (VMRs) of trace gases are retrieved by HEIPRO algorithm in this study (Heidelberg Profile, developed by IUP Heidelberg) (Frieß et al., 2006; 2011), coupled with the SCIATRAN radiative transfer scheme (Rozanov et al., 2002) as forward model. In general, the two-step approaches are implemented in the profiling algorithm, i.e., aerosol extinction coefficient (AEC) profile is firstly retrieved from measured O_4 DSCDs, which is then introduced into the radiative transfer model (RTM) as the aerosol scenario for the second step of trace gases profiles retrieval.

The vertical grid for retrieval was set to an altitude range of up to 3 km with a resolution of 100 m. The fixed a priori profiles were defined by an exponential decay function and

constructed with a priori profile error covariance matrix (Sa) of 100% for aerosol and NO_2, and 150% for BrO. The covariance length of 500 m for all profile inversions. The a priori extinction of aerosol and surface concentrations of NO_2 and BrO, were set to 0.1 km^{-1}, 1.0 and 0.01 ppbv, respectively. Parameters of surface albedo of 0.1, single-scattering albedo (SSA) of 0.95 and asymmetry factor for HG phase function of 0.72 were used for the radiative transfer simulations. The wavelength of profile retrieval is 360 nm for AEC, 350 nm for BrO and 432.5 nm for NO_2, respectively. And the retrieved aerosol scenario at 360 nm can be adapted for trace gas retrieval as input via the Ångström exponent within the algorithm scheme. Examples of aerosol extinction coefficient and trace gas profile retrievals are presented in Fig. S8. Afterwards, we set chi-square of 30 for O_4, 8 for HCHO and 50 for NO_2 as the thresholds, respectively, in order to ensure the reliability of the retrieval results. Besides, the results with the Degrees of Freedom for signal (DoF) lower than 2.5 for aerosol and 1.5 for trace gases were excluded from further discussion. After the filtering, the squares of the correlation coefficients (R^2) increased above 0.99 for aerosol and NO_2 and 0.93 for BrO, the slopes deviate from unity decreased below 4% for all species, as shown in Fig. S8d.

For SP-DOAS measurement, the spectral analysis yields directly integrated concentrations of atmospheric trace gases along the optical path and can be further converted to the average trace gas concentration via dividing by the length of the absorption path. The SP-DOAS measured spectra are analyzed using the DOASIS spectral fitting software (IUP in Heidelberg University, Germany). The fitting windows for O_3 are 262-272 nm, and absorption of SO_2 (Vandaele et al., 2009), HCHO (Meller and Moortgat et al., 2000) and O_2 (Bogumil et al, 2003) were considered (Gu et al., 2022).

Text S5. THAMO model simulation

Tropospheric Halogen Chemistry Model (THAMO; Mahajan et al., 2009; Tham et al., 2021) is used to show the response of BrO levels to a few critical factors (as shown in Fig. 2c), including NO_x, initial inorganic bromine level, and aerosol surface area (ASA). The observed (where available) and WRF-Chem simulated levels of O_3, NO, NO_2, CO, SO_2, HCHO, H_2O_2, CH_4, OH, HO_2, etc. are used to set up the simulation cases, including Standard, High_NO_x, High_INI, High_(INI+NO_x), High_ASA, High_(ASA+NO_x), and High_(INI+ASA+NO_x) cases. All available data points during the whole campaign are used to derive the average diurnal variation to constrain the THAMO simulations. Please refer to Table S2 for details.

An additional case (the Constrained case) was also conducted to use all available observation datasets (including BrO) to quantify the individual contribution of various processes to the overall loss of O_3 at HNI (as presented in Fig. 3a).

Text S6. WRF-Chem model simulation

In the present study, we employed a widely-used regional chemical transport model (WRF-Chem), incorporated with comprehensive bromine chemistry (Li et al., 2021a; Badia et al., 2019) ("Text S7. Reactive bromine sources and chemistry"), to investigate the potential influences of various sources on the abundance and impacts of reactive bromine species at HNI and the surrounding region (West Pacific and East Asia). We conducted eight cases (HAL, noSSA, lowORG, noANT, lrgGM, 7NO_x_HNI, wthDMS, and noBr; Table S3) to distinguish the impacts of (1) HOBr/$BrNO_2$/$BrONO_2$ heterogeneous efficiency (HAL v.s. noSSA), (2) biogenic source of organic bromine (HAL v.s. lowORG), (3) anthropogenic emission of reactive bromine (HAL v.s. noANT), (4) NO_x emission and abundance (HAL v.s. 7NOx_HNI), (5) bromine species uptake coefficients on SSA (HAL v.s. lrgGM), (6) all reactive bromine sources and processes on the abundance of bromine and other atmospheric compositions (HAL v.s. noBr), and (7) DMS source and chemistry on the simulated BrO levels (HAL v.s. wthDMS). Please note that Mass et al. (2021) reported that the organic Br emission (air-sea flux) in this region (along the coast of East Asia) could be significantly (7.75 to 37.75 times) larger than those reported by Ziska et al. (2013); therefore, in HAL, noSSA, and noANT cases, we applied a medium scaling factor (10) to the Ziska et al. (2013) organic Br emission.

The model setup follows our previous halogen modelling exercises in this region (Li et al., 2021a; 2021b). Table S5 summarizes the main settings adopted in this study. In particular, we adopted the sea-salt aerosol (SSA) emission parameterization proposed by Gong et al. (1997). In this parameterization, the SSA emission intensity (particles m^{-2} s^{-1} μm^{-1}) depends on the surface wind speed as shown in Eq1-2, in which u is the wind speed at 10m above sea level, r is the particle radius at 80% relative humidity. Such dependence of SSA emission on the wind speed is exponential: for a given radius of the particle, the SSA emission intensity at a wind speed of 20 m/s is >100 times stronger than that at a wind of 5 m s^{-1} (20^3.41=27322 v.s. 5^3.41=242). Note that the SSA emission intensity in other literatures (Gong et al., 2003; Jaeglé et al., 2011) also have similar exponential dependence of SSA on wind speed. The typhoon activity significantly stirs up the movement of tropospheric air and sea-air exchange, resulting in larger amounts of SSA in the MBL. The amplified amount of SSA subsequently leads to an outburst of reactive halogen species (including bromine) to the MBL, in a similar manner to the bromine explosion events reported in the Arctic.

$$\frac{dSSA}{dr} = 1.373u^{3.41}r^{-3}(1 + 0.057r^{1.05}) \times 10^{1.19e^{-B^2}} \quad \text{Eq(1)}$$

$$B = (0.38 - \log(r))/0.650 \quad \text{Eq(2)}$$

The loss of SSA mainly depends on the aerosol dry and wet depositions. The aerosol dry deposition is calculated online following the Wesely (1989) scheme. The in-cloud wet scavenging scheme follows the approach of Easter et al. (2004) and removes cloud-borne particles using the first-order loss rate of cloud water, while the below-cloud scavenging washes out aerosols by impaction and interception and was computed in the model based on Slinn (1984) parameterization. Thus, the grid-scale wet scavenging

depends on variables such as cloud water, ice, rain, snow, and graupel. Subgrid-scale wet scavenging is calculated using parameterized variables (updraft/downdraft mass flux, entrainment/detrainment rate, and precipitation rate) from the subgrid convection scheme used in the model (Grell and Devenyi, 2002). Typhoon intensity accelerates the formation of raindrops and increases SSA wet deposition.

We then compare the daytime average BrO mixing ratios in various WRF-Chem simulations, as shown in Fig. S5. Our WRF-Chem model (HAL case) is able to reproduce the key observed feature in the three periods, particularly the rapid enhancement in BrO mixing ratio under the influence of the typhoon. The comparisons between the main case (HAL) and other sensitivity simulations suggest that (1) the simulated BrO levels are very sensitive to the bromine heterogeneous reactions on SSA (noSSA v.s. HAL); (2) organic bromine species in seawater (chlorophyll-a related biogenic emissions) have small effects on the BrO levels at HNI in the MBL during the typhoon days but have some noticeable influence during other days (lowORG v.s. HAL); (3) the impact of the currently known continental anthropogenic bromine emissions on the BrO on HNI is very limited during the simulation period (noANT v.s. HAL); (4) our sensitivity tests (HAL v.s. 7NO_x_HNI, HAL v.s. lrgGM, HAL v.s. wthDMS) suggest that our main conclusion remains the same (typhoon→larger wind speed→more SSA→more bromine) regardless of the simulated levels of NO_x, BrO, and DMS; and (5) the heterogeneous uptake process of HOI on SSA (producing IBr and ICl) has limited contribution to the total bromine abundance in this region under typhoon influence. Please note that noANT refers to the case without anthropogenic bromine emissions.

In this study, we apply the WRF-Chem model to provide a first-order estimate of bromine abundance, its potential causes, and the corresponding impacts. Further studies are desired to quantify the relative contribution of different sources to the bromine abundance during the typhoon period, to estimate the impact (or the range of the impact) of bromine species on atmospheric chemistry, and to assess the policy-relevance of halogen chemistry in air quality regulation.

Text S7. Reactive bromine sources and chemistry

The sources of reactive bromine species consist of (1) direct emission of organic bromine species ($CHBr_3$, CH_2Br_2, $CHBr_2Cl$, $CHBrCl_2$, etc.) from the ocean surface, closely related to the biogenic activity (the level of chlorophyll-α) in the surface ocean (Ordóñez et al., 2012); (2) gaseous inorganic bromine species (e.g., Br_2 and BrCl) release from the heterogeneous processing of bromine-containing species on sea-salt aerosol (R1; Fernandez et al., 2014); (3) direct emission of reactive bromine species (e.g., HBr and Br_2) from anthropogenic activities (Li et al., 2021a); (4) other sources, e.g., volcano and polar processes (e.g., Fernandez et al., 2019), that are less relevant to mid-latitude regions.

$$HOBr/BrNO_2/BrONO_2 + SSA \rightarrow aBr_2 + bBrCl \quad (2a+b \geqq 1) \qquad (R1)$$

The first-order reaction rate of $HOBr/BrNO_2/BrONO_2$ heterogeneous uptake on SSA is as follows:

$$\text{Reaction rate} = \frac{\gamma CS}{4} \qquad \text{Eq(3)}$$

in which γ is the dimensionless reactive uptake coefficient, C is the molecular speed of the uptake gas species (depending on air temperature; cm s^{-1}), and S is the aerosol surface area (cm^2 cm^{-3}).

Reactive bromine species actively participate in tropospheric chemistry. With the presence of VOC and NO_x (in polluted regions), the Br atom is transformed to $BrNO_2$ (R2) or oxidises VOC species leading to the production of OH, O_3, and NO_3 (R3 - R9). In clean and semi-polluted environments (e.g., HNI), the Br atom mostly reacts with O_3 and forms BrO, an important indicator of reactive bromine chemistry in the troposphere (R10). The reduced amount of O_3 decreases the production of OH (R11-12). BrO reacts with HO_2 (forming HOBr; R13) or NO_2 (forming $BrONO_2$; R14) depending on their relative abundance. HOBr photolyzes and forms Br atom and OH radical (R15); the combination of R11 and R13 transforms HO_2 to OH and increases OH levels in some regions (ref). HBr, $BrNO_2$, and $BrONO_2$ also recycle bromine species through heterogeneous reactions (R16-R17).

$$Br + NO_2 \rightarrow BrNO_2 \qquad (R2)$$
$$Br + VOC \rightarrow RO_2 + HBr \qquad (R3)$$
$$RO_2 + NO \rightarrow NO_2 + RO \qquad (R4)$$
$$RO + O_2 \rightarrow HO_2 + \text{carbonyls} \qquad (R5)$$
$$HO_2 + NO \rightarrow NO_2 + OH \qquad (R6)$$
$$NO_2 + hv \rightarrow NO + O(^3P) \qquad (R7)$$
$$O(^3P) + O_2 \rightarrow O_3 \qquad (R8)$$
$$NO_2 + O_3 \rightarrow NO_3 \qquad (R9)$$
$$Br + O_3 \rightarrow BrO \qquad (R10)$$
$$O_3 + hv \rightarrow O^1D + O_2 \qquad (R11)$$
$$O^1D + H_2O \rightarrow 2OH \qquad (R12)$$
$$BrO + HO_2 \rightarrow HOBr \qquad (R13)$$
$$BrO + NO_2 \rightarrow BrONO_2 \qquad (R14)$$
$$HOBr + hv \rightarrow Br + OH \qquad (R15)$$
$$N_2O_5 + HBr (+\text{aerosol}) \rightarrow BrNO_2 + HNO_3 \qquad (R16)$$
$$BrNO_2/BrONO_2 + H_2O/HCl (+\text{aerosol}) \rightarrow HOBr/BrCl + HONO/HNO_3 \qquad (R17)$$

Text S8. Possibility of enhancement in reactive chlorine and iodine abundance due to typhoon activity

The activation of reactive chlorine and iodine during the SSA increase is also worth to be discussed. However, the spectrometer equipped to the MAX-DOAS instrument in this study can only record the light signal between 290 and 470 nm. The spectral fitting range for reactive chlorine and iodine, like ClO and IO, is usually around 300-320 nm

(Bobrowski et al., 2007) and 420-450 nm (Gómez Martín et al., 2013) by utilizing passive DOAS method measuring scattered sunlight, which are not in the spectral range of the spectrometer with the best performance. So, we are not able to detect the related chlorine and iodine species, which is unfavorable to further investigate their impacts related to typhoon process based on the measurements.

In principle, enhanced release of chlorine is indeed possible from enhanced SSA concentration, considering that the acid displacement process (HNO_3+SSA->HCl) is one of the major sources of reactive chlorine gases in the MBL. However, the abundance of chloride in SSA is generally treated as saturated and the limiting factor of this process is the amount of HNO_3, instead of the amount of SSA. Meanwhile, the activation of bromine gases from SSA (HOBr/$BrNO_2$/$BrNO_3$ + SSA -> $0.65Br_2$ + 0.35BrCl, as used in our model) is exponentially enhanced with the increased amount of SSA. Note that this bromine-induced chlorine activation (i.e., BrCl production) is a minor term compared to the acid displacement process. Therefore, we expect that the enhancement in reactive chlorine species during the typhoon period to be smaller than that in bromine species. Future studies, particularly field observations, are recommended to further investigate the potential enhancement in chlorine activation due to typhoon activities.

The main source of reactive iodine in the MBL is the O_3 deposition onto the sea surface, releasing I_2 and HOI to the atmosphere. The heterogeneous process of reactive iodine involving SSA is actually a recycling one instead of a net source or sink (Li et al., 2022; Saiz-Lopez et al., 2015, 2023; Barrera et al., 2023). Therefore, we expect that the increase in SSA will not substantially affect the source of reactive iodine. Future studies are recommended to look into this aspect.

Text S9. Satellite-derived Chl-α data

In addition, chlorophyll-a (Chl-a) concentrations of the surrounding sea areas derived from satellite observation were acquired for the relevant BrO measurements, as listed in Table S1. Here, we used the 8-day composite L3 products of chlorophyll-a concentration derived from the Moderate Resolution Imaging Spectroradiometer (MODIS) on the polar-orbiting satellite TERRA platform for previous MBL measurements (data available from: https://oceancolor.gsfc.nasa.gov/l3/). For HNI measurements, chlorophyll-a concentration product of the Himawari-8 geostationary meteorological satellite was selected for higher temporal resolution (data available by request from: https://www.eorc.jaxa.jp/ptree/index.html). Chlorophyll-a concentration data were averaged for measurement locations (in-situ measurements) or centre of areas (cruise measurement) with a radius of 20 km and the corresponding measurement period. The calculated daily Chl-a data and corresponding BrO VMR are presented in Fig. 2b in the main text and Fig. S4 in the supplement.

Text S10. Typhoon "Ampil"

During the observational period, a typhoon was formed in the northwest Pacific and named Ampil at 12:00 UTC on July 18, 2018. It passed over Okinawa Island from July 20 to 21, thereafter weakened slightly while crossing the ECS before making landfall in Shanghai, China, at approximately 12:30 on July 22 with a maximum wind speed of 28 m s^{-1} in the center. It continued moving northwest and arrived at the North China Plain in the afternoon of July 23. By July 24 evening, Ampil weakened and became an extratropical cyclone with decreased intensity. The full pathway of Ampil movement can be found in Fig. S6. The shortest distance of the Ampil track from the HNI site is only 20 km around 09:00 LT on July 22, causing the extremely high wind speed of 36.5 m/s at HNI about 06:50 LT.

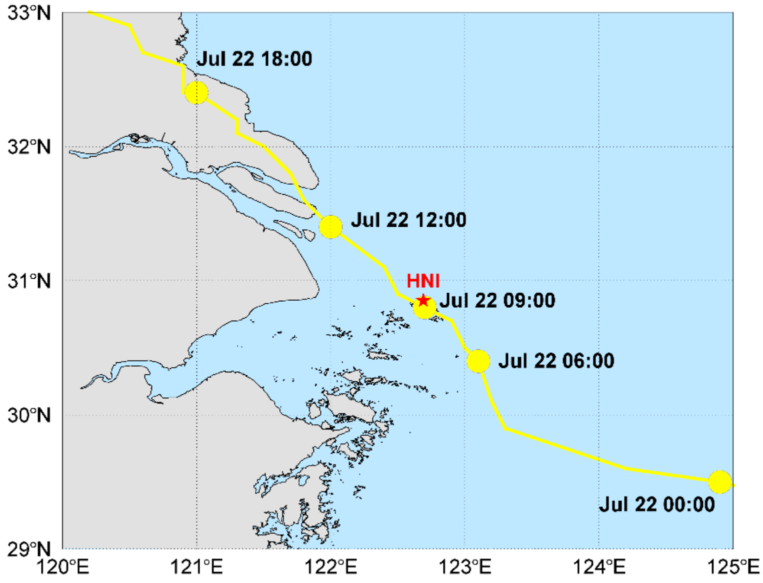

Figure S1 | Location of the measurement site of Huaniao Island in the East China Sea, China together with the track of typhoon Ampil in 2018. Information on the moving position of Typhoon Ampil was obtained from the Typhoon and Marine Forecasting Center of the China Meteorological Administration (http://typhoon.nmc.cn).

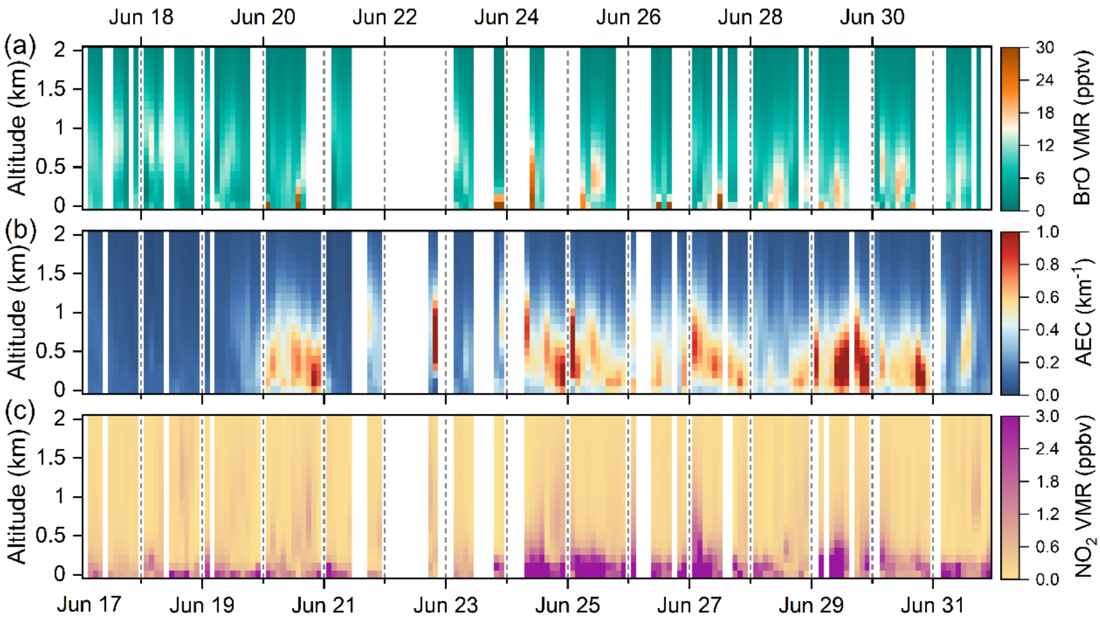

Figure S2 | Time series of the daytime BrO, aerosol extinction coefficient and NO_2 vertical distribution, observed by MAX-DOAS in HNI site, China.

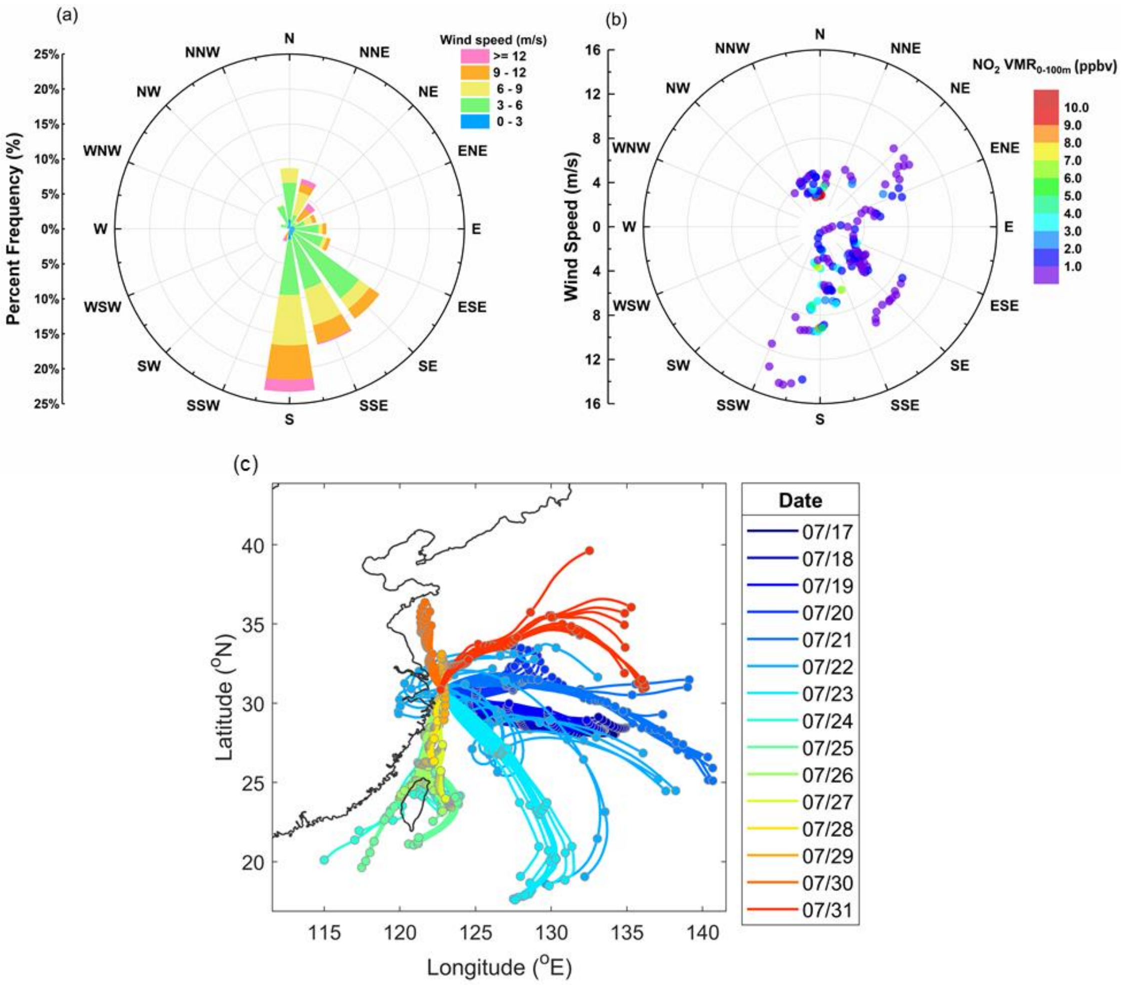

Figure S3 | Potential source region of the air mass arriving at HNI. (a) Wind rose during the observation, (b) the dependency of surface NO_2 VMR retrieved from MAX-DOAS measurements at daytime, and (c) back-trajectories of the air masses reaching HNI for each day during the measurement (generated via the MeteoInfoMap software (Wang, 2014) based on meteorological data from the Global Data Assimilation System (ftp://arlftp.arlhq.noaa.gov/pub/archives/gdas1/)).

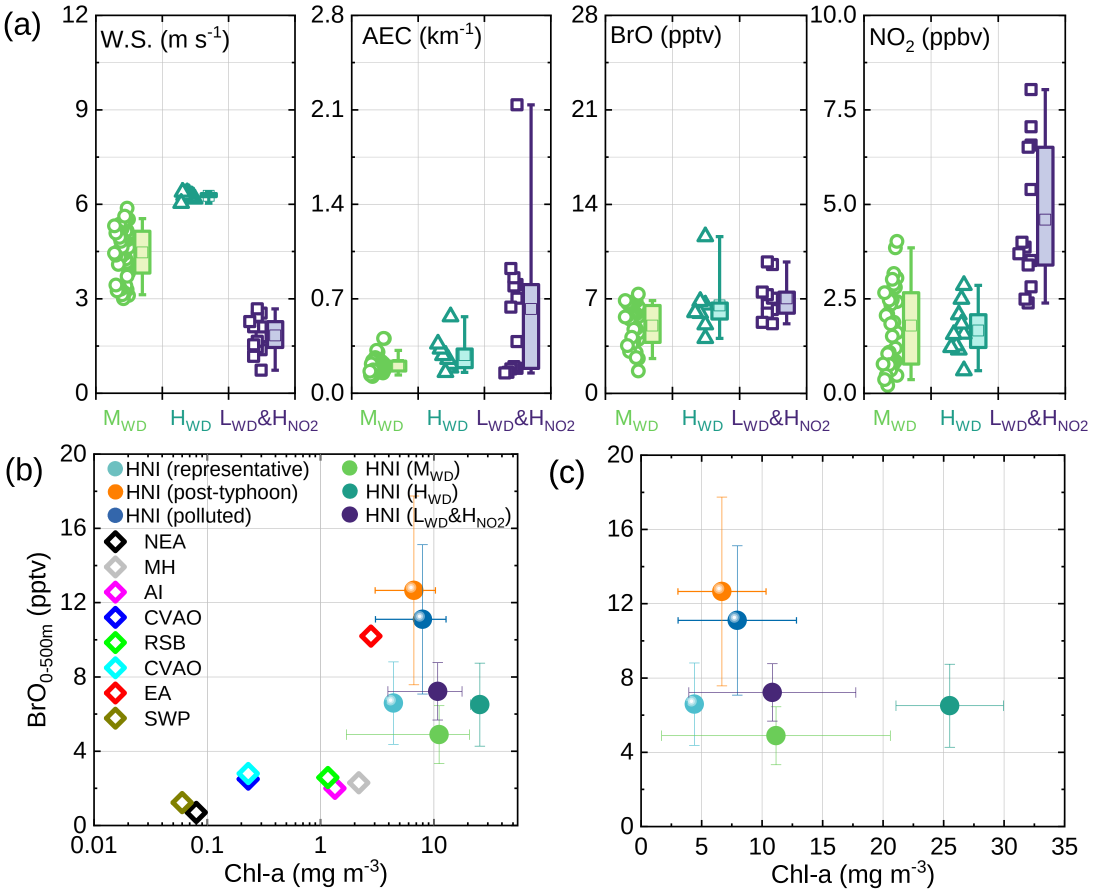

Figure S4 | Relationship between observed BrO VMR with wind speed, AEC, NO_2 and Chl-a at HNI in the spring 2018. (a) statistics for the wind speed, AEC, BrO and NO_2 on different characterized conditions of: (1) middle wind speed (3-6 m/s, "M_{WD}"), (2) high wind speed (> 6m/s, "H_{WD}") and (3) lower wind speed (≤ 3 m/s) combined with high NO_2 levels (> 2 ppbv) ("L_{WD}&H_{NO2}") during spring 2018; (b) and (c) observed BrO VMR with Chl-a for previous observation in middle latitude MBL worldwide in addition to HNI results in spring and summer 2018.

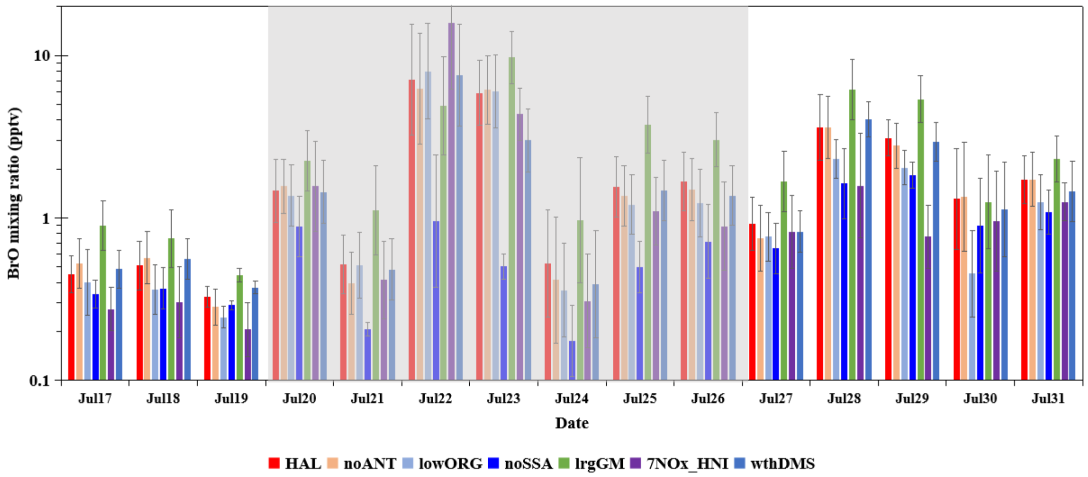

Figure S5 | Comparison of simulated BrO in different WRF-Chem cases (HAL, noSSA, lowORG, noANT, lrgGM, 7NOx_HNI, wthDMS) at HNI during the field campaign. The gray-shaded area indicates the days under typhoon influence. Similar to the observation at HNI, WRF-Chem results also showed the lowest BrO mixing ratios during representative days (Jul 17 to 19) and a rapid increase in BrO with the influence of typhoon activities in this area (July 20-26), while maintaining elevated levels after the typhoon including the polluted periods (July 27-30).

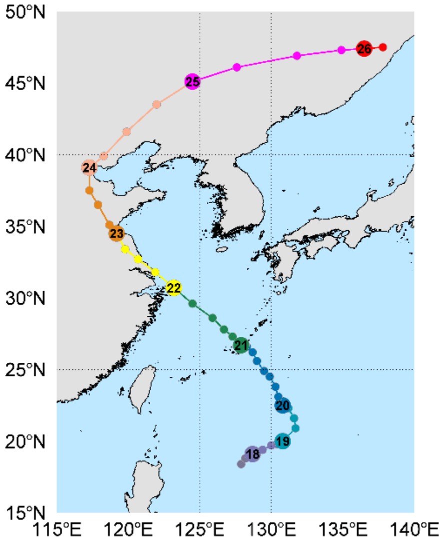

Figure S6 | Track map of Ampil in 2018. Information on the moving position of Typhoon Ampil was obtained from the Typhoon and Marine Forecasting Center of the China Meteorological Administration (http://typhoon.nmc.cn).

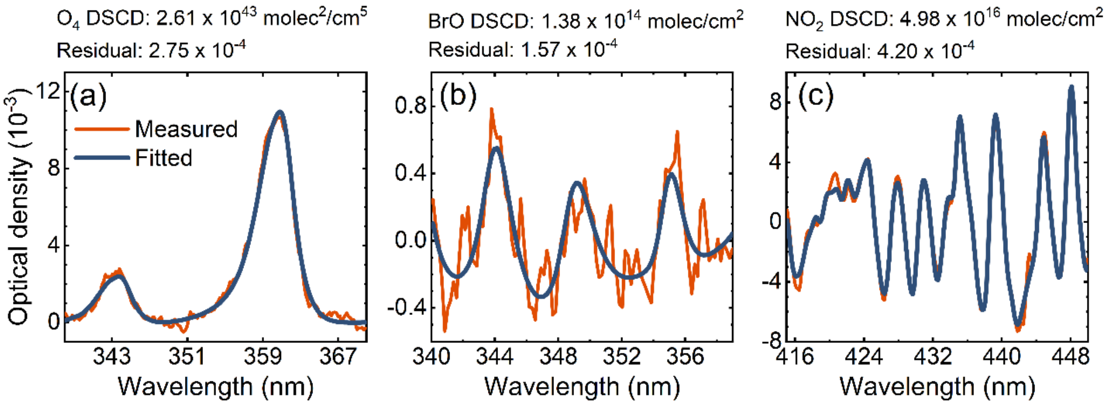

Figure S7 | Examples of spectral fitting for (a) O_4, (b) BrO and (c) NO_2 of spectrum measured at an elevation angle of 0° at 07:02 LT on July 19, 2018. The fitted DSCDs and RMS are given in the individual subpanels.

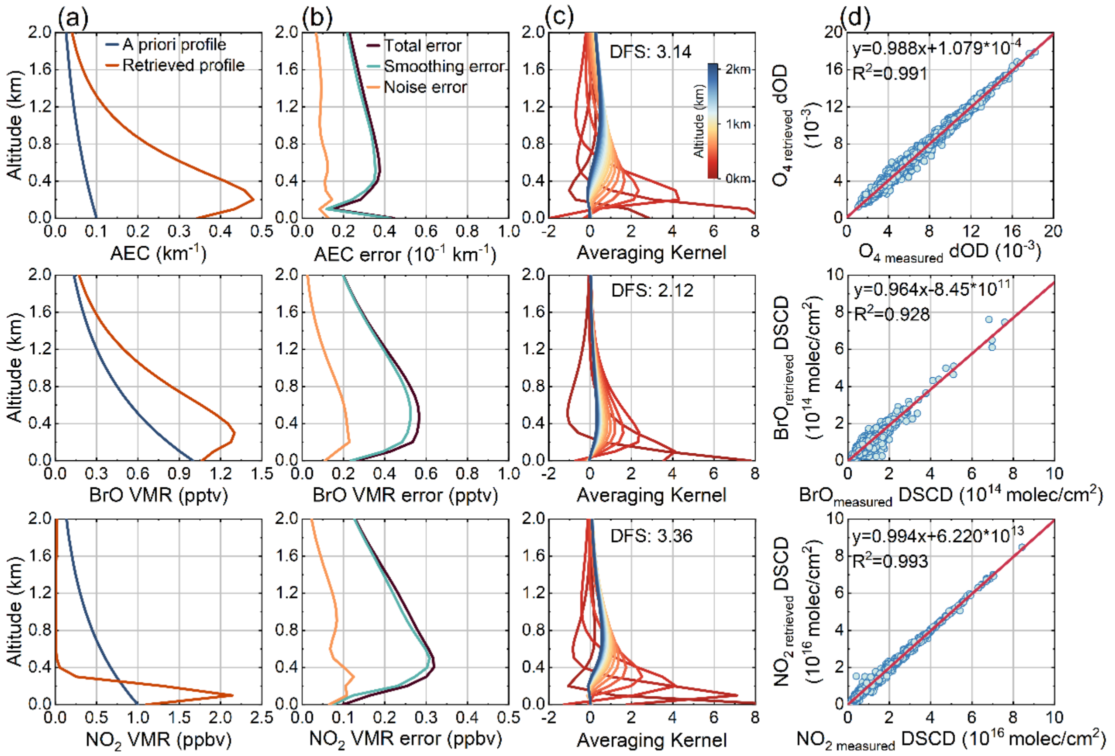

Figure S8 | Examples of retrievals from MAX-DOAS measurements on July 28, 2018 around 09:55 LT. (a) A priori profiles and retrieved profiles; (b) The retrieval errors; (c) The average kernels with information on the degree of freedom. (d) Scatter plots of measured DSCDs (ODs) against modelled DSCDs (ODs) during the whole measurement period. The squares of the correlation coefficients (R^2) and the slopes derived from the linear regressions (red lines) are given in each subplot. The upper, middle and lower panels indicate the aerosol extinction, BrO and NO_2 retrievals, respectively.

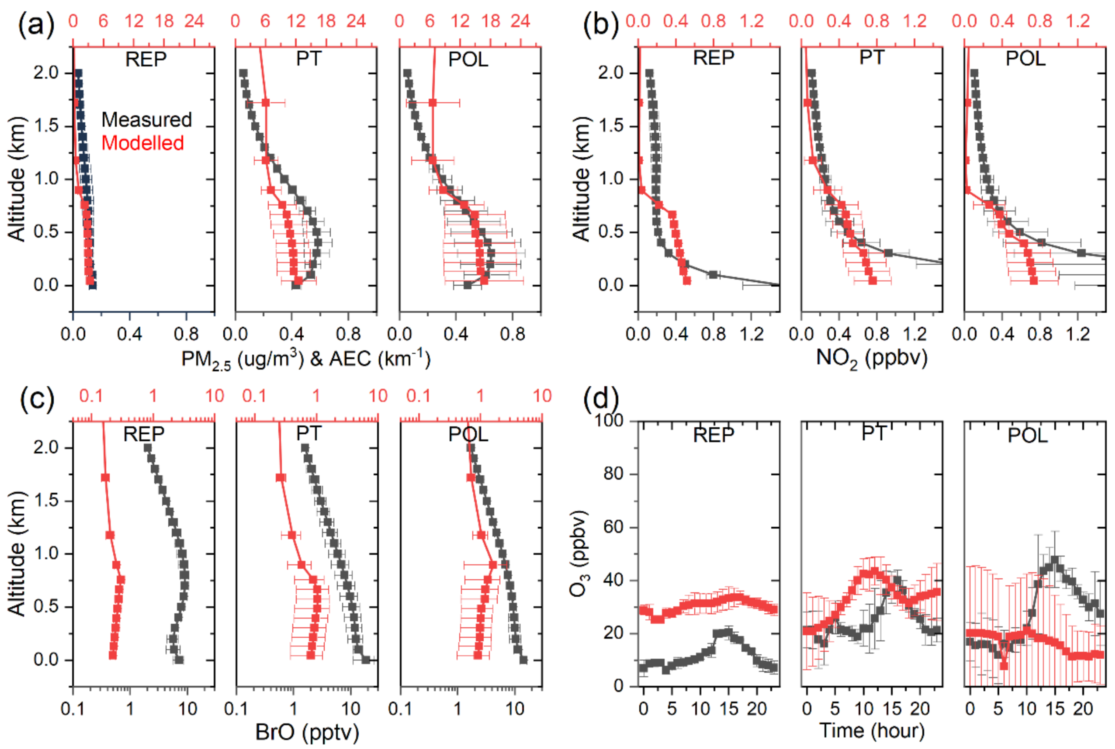

Figure S9 | Comparison of observed (MAX-DOAS) and simulated NO_2 (profile), BrO (profile), and O_3 (surface mixing ratio) at HNI. A plot with observed AEC profile and simulated $PM_{2.5}$ profile is also included to show that WRF-Chem is able to reproduce the key aerosol extinction characteristics, particularly the changes from representative (REP) condition to the post-typhoon (PT) and to the polluted condition (POL). The solid blocks indicate the mean, while the error bars show the standard deviation.

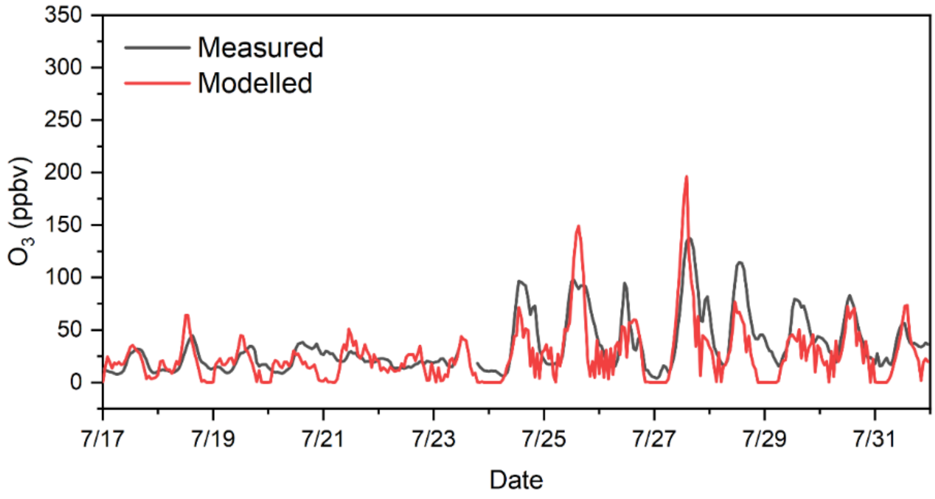

Figure S10 | Measured and simulated (WRF-Chem) O_3 mixing ratio in Shanghai from July 17 to July 31 in 2018.

Table S1 | Summary of previous observed daytime BrO in MBL and corresponding chlorophyll-a (Chl-a) concentration of the surrounding sea areas.

References	Location	Time	BrO (pptv)	NO_x (ppbv)	Chl-a (mg/m^3)
Leser et al., 2003	13 W, 30-37 N; Northeast Atlantic, NEA	2000/10/6-2000/10/7	0.7±0.2	/	0.08
Saiz-Lopez et al., 2004	9.9 W, 53.33 N; Mace Head, MH	Aug. 2002	2.3	<0.5<br>NO_2	2.18
Keene et al., 2007	70.62 W, 42.90 N; Appledore Island, AI	2004/7/6-2004/8/12	< 2	/	1.34
Read et al., 2008<br>Mahajan et al., 2010	24.87 W, 16.85 N; Cape Verde Atmospheric Observatory, CVAO	2006/11-2007/6	2.5±1.1; mean maxima<br>5.6±1; maximum	0.003±0.001; NO	0.23
Mahajan et al., 2009	4.0 W, 48.7 N; Roscoff Station Biologique, RSB	2006/9/6-2006/9/22	2.58	DL to 4.8±0.5; NO_2	1.16
Martin et al., 2009	17-17.4 W, 18 N; East Atlantic, EA	2007/2/18	10.2±3.7; peak value	/	2.79
Le Breton et al., 2017	134.5 E, 7.4 N - 144.8 E, 13.5 N; Southwest Pacific, SWP	2014/1/18	1.23	/	0.06
This study	30.85 N, 122.69 E; Huaniao Island, HNI	2018/7/17-2018/7/31	9.7±4.2	1.88±2.27	6.05

Table S2 | Design of THAMO box model simulations

Cases	O_3	NOx	SO_2, CO, OH, HO_2, CO, HCHO, CH_4	Initial BrOy	BrO	Aerosol Surface Area
**Standard**	Observation	Observation	WRF-Chem simulation	5 pptv	Produced in THAMO	5.E-7 cm^{-2} cm^{-3}
**High_NO_x**	Observation	Double of that in Standard case.	WRF-Chem simulation	5 pptv	Produced in THAMO	5.E-7 cm^{-2} cm^{-3}
**High_INI**	Observation	Observation	WRF-Chem simulation	15 pptv	Produced in THAMO	5.E-7 cm^{-2} cm^{-3}
**High_(INI+NOx)**	Observation	Double of that in Standard case	WRF-Chem simulation	15 pptv	Produced in THAMO	5.E-7 cm^{-2} cm^{-3}
**High_ASA**	Observation	Observation	WRF-Chem simulation	5 pptv	Produced in THAMO	6.5E-7 cm^{-2} cm^{-3}
**High_(ASA+NOx)**	Observation	Double of that in Standard case	WRF-Chem simulation	5 pptv	Produced in THAMO	6.5E-7 cm^{-2} cm^{-3}
**High_(INI+ASA+NOx)**	Observation	Double of that in Standard case	WRF-Chem simulation	15 pptv	Produced in THAMO	6.5E-7 cm^{-2} cm^{-3}
**Constrained**	Observation	Observation	WRF-Chem simulation	0 pptv	Observation	5.E-7 cm^{-2} cm^{-3}

Table S3 | Design of WRF-Chem regional model simulations

Cases	Continental air pollutant emission	Shipping emission in East China Sea	Anthropogenic Br emission	Oceanic Br emission	Uptake coefficient of $HOBr/BrNO_2/BrNO_3$ on SSA and products	Other Br chemistry	DMS sources and chemistry
HAL	MEIC	Fan et al. (2016); Yuan et al. (2023)	Li et al. (2021a)	Badia et al. (2019); 10 times of Ziska et al. (2013)	0.15 ($0.65Br_2$+0.35BrCl)	Badia et al. (2019); Li et al. (2021a)	None
noSSA	MEIC	Fan et al. (2016); Yuan et al. (2023)	Li et al. (2021a)	Badia et al. (2019); 10 times of Ziska et al. (2013)	None	Badia et al. (2019); Li et al. (2021a)	None
lowORG	MEIC	Fan et al. (2016); Yuan et al. (2023)	Li et al. (2021a)	Badia et al. (2019); Ziska et al. (2013)	0.15 ($0.65Br_2$+0.35BrCl)	Badia et al. (2019); Li et al. (2021a)	None
noANT	MEIC	Fan et al. (2016); Yuan et al. (2023)	None	Badia et al. (2019); 10 times of Ziska et al. (2013)	0.15 ($0.65Br_2$+0.35BrCl)	Badia et al. (2019); Li et al. (2021a[a])	None
lrgGM	MEIC	Fan et al. (2016); Yuan et al. (2023)	Li et al. (2021a)	Badia et al. (2019); 10 times of Ziska et al. (2013)	0.3 ($0.65Br_2$+0.35BrCl)	Badia et al. (2019); Li et al. (2021a)	None
7NOx_HNI	MEIC	7 times of Fan et al. (2016) & Yuan et al. (2023) in HNI; Fan et al. (2016) & Yuan et al. (2023) in the rest areas	Li et al. (2021a)	Badia et al. (2019); 10 times of Ziska et al. (2013)	0.15 ($0.65Br_2$+0.35BrCl)	Badia et al. (2019); Li et al. (2021a)	None
noBr	MEIC	Fan et al. (2016); Yuan et al. (2023)	None	None	None	None	None
wthDMS	MEIC	Fan et al. (2016); Yuan et al. (2023)	Li et al. (2021a)	Badia et al. (2019); 10 times of Ziska et al. (2013)	0.15 ($0.65Br_2$+0.35BrCl)	Badia et al. (2019); Li et al. (2021a)	Li S. et al., (2020) Veres et al., (2020)

Table S4 | Summary of DOAS spectral fitting configuration for O_4, BrO and NO_2 DSCDs retrieval.

Cross Section		O_4	BrO	NO_2
O_4	Thalman and Volkamer (2013), 293 K	√	√	√
O_3	Serdyuchenko et al. (2014), 223 K, 243 K	√	√	Only 223 K
HCHO	Meller and Moortgat (2000), 298 K	√	√	
BrO	Wilmouth et al. (1999), 298 K	√	√	
NO_2	Vandaele et al. (1998), 298 K, 220 K	√	√	√
H_2O	Rothman et al., (2010), 296 K			√
SO_2	Bogumil et al. (2003), 293 K		√	
Ring	Calculated by QDOAS software	√	√	√
Fitting windows		338-370 nm	340-359 nm	415-450 nm
Polynomial degree		5th	3th	4th
Intensity offset		Constant	None	Order 1

Table S5 | Model settings and input data adopted in WRF-Chem

**Category**	**Options**
Radiation	RRTMG (Iacono et al. 2008)
Land-surface model	Noah land surface model (Chen and Dudhia, 2001)
Microphysics scheme	Morrison double-moment scheme (Morrison et al., 2008)
PBL scheme	Quasi-Normal Scale Elimination PBL scheme (Sukoriansky, et al. 2005)
Gas chemistry	MOZART (Emmons et al. 2010; Badia et al. 2019; Li et al., 2021a)
Aerosol chemistry	MOSAIC (Zaveri et al. 2008; Badia et al. 2019; Li et al., 2021a)
Photolysis	FTUV (Tie et al. 2003; Badia et al. 2019)
Sea-salt aerosol emission	Gong et al., 1997; Archer-Nicholls et al., 2014
Anthropogenic emission	MEIC (www.meicmodel.org)
Shipping emission	Fan et al., 2016; Yuan et al., 2023
Biogenic emission	MEGAN (Guenther et al., 2006)
Biomass burning emission	FINN (Wiedinmyer et al., 2011)
Initial and Boundary condition	CAM-Chem global model output (Li et al., 2022)
Horizontal resolution	27 km
Simulation period	May 15, 2018 to July 31, 2018 with the first 60 days as spin-up

References (sorted by the order of appearance)

Zhang, R., Wang, S., Zhang, S., Xue, R., Zhu, J., and Zhou, B. (2022). MAX-DOAS observation in the midlatitude marine boundary layer: Influences of typhoon forced air mass. J. Environ. Sci. 120, 63-73.

Li, X., Chen, M., Le, H.P., Wang, F., Guo, Z., Iinuma, Y., et al. (2016). Atmospheric outflow of $PM_{2.5}$ saccharides from megacity Shanghai to East China Sea: Impact of biological and biomass burning sources. Atmos. Environ. 143, 1–14.

Nakamura, T., Matsumoto, K., Uematsu, M. (2005). Chemical characteristics of aerosols transported from Asia to the East China Sea: an evaluation of anthropogenic combined nitrogen deposition in autumn. Atmos. Environ. 39, 1749–1758.

Pani, S.K., Lee, C.T., Chou, C.C.K., Shimada, K., Hatakeyama, S., Takami, A., et al. (2017). Chemical Characterization of Wintertime Aerosols over Islands and Mountains in East Asia: Impacts of the Continental Asian Outflow. Aerosol Air Qual. Res. 17, 3006–3036.

Guo, T., Lin, T., Li, Y., Wu, Z., Jiang, Y., Guo, Z. (2019). Occurrence, gas-particle partitioning, and sources of polybrominated diphenyl ethers in the atmosphere over the Yangtze River Estuary, East China Sea. Sci. Total Environ. 693, 133538.

Yang, T., Chen, Y., Zhou, S., Li, H., Wang, F. (2020). Solubilities and deposition fluxes of atmospheric Fe and Cu over the Northwest Pacific and its marginal seas. Atmos. Environ. 239, 117763.

Wang, F., Feng, T., Guo, Z., Li, Y., Lin, T., Rose, N. L. (2019). Sources and dry deposition of carbonaceous aerosols over the coastal East China Sea: Implications for anthropogenic pollutant pathways and deposition. Environ. Pollut. 245, 771–779.

Saiz-Lopez, A. J. M. C., Plane, J. M. C., & Shillito, J. A. (2004). Bromine oxide in the mid-latitude marine boundary layer. Geophysical Research Letters, 31(3), L03111.

Leser, H., Hönninger, G., & Platt, U. (2003). MAX-DOAS measurements of BrO and NO2 in the marine boundary layer. Geophysical Research Letters, 30(10), 1537.

Read, K. A., Mahajan, A. S., Carpenter, L. J., Evans, M. J., Faria, B. V., Heard, D. E., ... & Plane, J. M. (2008). Extensive halogen-mediated ozone destruction over the tropical Atlantic Ocean. Nature, 453(7199), 1232-1235.

Mahajan, A. S., Plane, J. M. C., Oetjen, H., Mendes, L., Saunders, R. W., Saiz-Lopez, A., ... & McFiggans, G. B. (2010). Measurement and modelling of tropospheric reactive halogen species over the tropical Atlantic Ocean. Atmospheric Chemistry and Physics, 10(10), 4611-4624.

Keene, W. C., Stutz, J., Pszenny, A. A., Maben, J. R., Fischer, E. V., Smith, A. M., von Glasow, R., Pechtl, S., Sive, B. C., and Varner, R. K. (2007). Inorganic chlorine and bromine in coastal New England air during summer. Journal of Geophysical Research: Atmospheres, 112(D10), D10S12.

Le Breton, M., Bannan, T. J., Shallcross, D. E., Khan, M. A., Evans, M. J., Lee, J., ... & Percival, C. J. (2017). Enhanced ozone loss by active inorganic bromine chemistry in the tropical troposphere. Atmospheric Environment, 155, 21-28.

Mahajan, A. S., Oetjen, H., Lee, J. D., Saiz-Lopez, A., McFiggans, G. B., & Plane, J. M. (2009). High bromine oxide concentrations in the semi-polluted boundary layer. Atmospheric Environment, 43(25), 3811-3818.

Martin, M., Pöhler, D., Seitz, K., Sinreich, R., & Platt, U. (2009). BrO measurements over the eastern North-Atlantic. Atmospheric Chemistry and Physics, 9(24), 9545-9554.

Platt, U., and Stutz, J. (2008). Differential Optical Absorption Spectroscopy, Springer, Berlin-Heidelberg, 2008.

Zhang, J., Wang, S., Guo, Y., Zhang, R., Qin, X., Huang, K., Wang, D., Fu, Q., Wang, J., and Zhou, B. (2018). Aerosol vertical profile retrieved from ground-based MAX-DOAS observation and characteristic distribution during wintertime in Shanghai, China. Atmos. Environ. 192, 193-205.

Cheng, Y., Wang, S., Zhu, J., Guo, Y., Zhang, R., Liu, Y., Zhang, Y., Yu, Q., Ma, W., and Zhou, B. (2019). Surveillance of SO2 and NO2 from ship emissions by MAX-DOAS measurements and the implications regarding fuel sulfur content compliance. Atmos. Chem. Phys. 19, 13611–13626.

Wang, S., Nan, J., Shi, C., Fu, Q., Gao, S., Wang, D., Cui, H., Saiz-Lopez, A. and Zhou, B. (2015). Atmospheric ammonia and its impacts on regional air quality over the megacity of Shanghai, China. Sci. Rep. 5, 1-13.

Guo, Y., Wang, S., Zhu, J., Zhang, R., Gao, S., Saiz-Lopez, A., and Zhou, B. (2021). Atmospheric formaldehyde, glyoxal and their relations to ozone pollution under low- and high-NOx regimes in summertime Shanghai, China. Atmos. Res. 258, 105635.

Wagner, T., Beirle, S., Brauers, T., Deutschmann, T., Frieß, U., Hak, C., et al. (2011). Inversion of tropospheric profiles of aerosol extinction and HCHO and NO_2 mixing ratios from MAX-DOAS observations in Milano during the summer of 2003 and comparison with independent data sets. Atmos. Meas. Tech. 4, 2685-2715.

Wagner, T., Beirle, S., Remmers, J., Shaiganfar, R., and Wang, Y. (2016). Absolute calibration of the colour index and O_4 absorption derived from Multi AXis (MAX-)DOAS measurements and their application to a standardised cloud classification algorithm. Atmos. Meas. Tech. 9, 4803-4823.

Frieß, U., Monks, P.S., Remedios, J.J., Rozanov, A., Sinreich, R., Wagner, T., and Platt, U. (2006). MAX-DOAS O4 measurements: A new technique to derive information on atmospheric aerosols: 2. Modeling studies. J. Geophys. Res. 111, D14203.

Frieß, U., Sihler, H., Sander, R., Pöhler, D., Yilmaz, S., and Platt, U. (2011). The vertical distribution of BrO and aerosols in the Arctic: Measurements by active and passive differential optical absorption spectroscopy. J. Geophys. Res. 116, D00R04.

Rozanov, V.V., Buchwitz, M., Eichmann, K.U., de Beek, R., and Burrows, J.P. (2002). SCIATRAN - A new radiative transfer model for geophysical applications in the 240-2400 nm spectral region: The pseudo-spherical version. Adv. Space Res. 29, 1831-1835.

Vandaele, A.C., Hermans, C., and Fally, S. (2009). Fourier transform measurements of SO_2 absorption cross sections: II. Temperature dependence in the 29000-44000 cm^{-1} (227-345 nm) region. J. Quant. Spectrosc. Radiat. Transfer 110, 2115-2126.

Meller, R. and Moortgat, G.K. Temperature dependence of the absorption cross sections of formaldehyde between 223 and 323 K in the wavelength range 225–375 nm. J. Geophys. Res.: Atmos. 105, 7089-7101 (2000).

Bogumil, K., Orphal, J., Homann, T., Voigt, S., Spietz, P., Fleischmann, O.C., et al. (2003). Measurements of molecular absorption spectra with the SCIAMACHY pre-flight model: instrument characterization and reference data for atmospheric remote-sensing in the 230-2380 nm region. J. Photochem. Photobiol. A 157, 167-184.

Gu, C., Wang, S., Zhu, J., Wu, S., Duan, Y., Gao, S., and Zhou, B. (2022). Investigation on the urban ambient isoprene and its oxidation processes. Atmos. Environ. 270, 118870.

Tham, Y.J., He, X.C., Li, Q., Cuevas, C.A., Shen, J., Kalliokoski, J., Yan, C., Iyer, S., Lehmusjärvi, T., Jang, S. and Thakur, R.C. (2021). Direct field evidence of autocatalytic iodine release from atmospheric aerosol. Proc. Natl Acad. Sci. USA 118, e2009951118.

Li, Q., Fu, X., Peng, X., Wang, W., Badia, A., Fernandez, R.P., Cuevas, C.A., Mu, Y., Chen, J., Jimenez, J.L. and Wang, T. (2021a). Halogens enhance haze pollution in China. Environmental Science & Technology, 55(20), 13625-13637.

Badia, A., Reeves, C. E., Baker, A. R., Saiz-Lopez, A., Volkamer, R., Koenig, T. K., Apel, E. C., Hornbrook, R. S., Carpenter, L. J., Andrews, S. J., Sherwen, T. and von Glasow, R. (2019). Importance of reactive halogens in the tropical marine atmosphere: a regional modelling study using WRF-Chem. Atmos. Chem. Phys. 19, 3161–3189.

Maas, J., Tegtmeier, S., Jia, Y., Quack, B., Durgadoo, J.V. and Biastoch, A. (2021). Simulations of anthropogenic bromoform indicate high emissions at the coast of East Asia. Atmos. Chem. Phys. 21, 4103-4121.

Ziska, F., Quack, B., Abrahamsson, K., Archer, S. D., Atlas, E., Bell, T., Butler, J. H., Carpenter, L. J., Jones, C. E., Harris, N. R. P., Hepach, H., Heumann, K. G., Hughes, C., Kuss, J., Krüger, K., Liss, P., Moore, R. M., Orlikowska, A., Raimund, S., Reeves, C. E., Reifenhäuser, W., Robinson, A. D., Schall, C., Tanhua, T., Tegtmeier, S., Turner, S., Wang, L., Wallace, D., Williams, J., Yamamoto, H., Yvon-Lewis, S., and Yokouchi, Y. (2013). Global sea-to-air flux climatology for bromoform, dibromomethane and methyl iodide. Atmos. Chem. Phys. 13, 8915–8934.

Li, Q., Badia, A., Fernandez, R. P., Mahajan, A. S., López-Noreña, A. I., Zhang, Y., Wang, S., Puliafito, E., Cuevas, C. A., Saiz-Lopez, A. (2021b). Chemical interactions between ship-originated air pollutants and ocean-emitted halogens. J. Geophys. Res.: Atmos. 126, e2020JD034175.

Gong, S. L., Barrie, L. A., and Blanchet, J.-P. (1997). Modeling sea-salt aerosols in the atmosphere: 1. Model development, J. Geophys. Res. 102, 3805– 3818.

Gong, S. L. (2003). A parameterization of sea-salt aerosol source function for sub- and super-micron particles. Global Biogeochem. Cycles 17, 1097.

Jaeglé, L., Quinn, P. K., Bates, T. S., Alexander, B., and Lin, J.-T. (2011). Global distribution of sea salt aerosols: new constraints from in situ and remote sensing observations. Atmos. Chem. Phys. 11, 3137–3157.

Wesely, M. (1989). Parameterization of surface resistances to gaseous dry deposition in regional-scale numerical models. Atmos. Environ. 23, 1293–1304.

Easter, R. C., Ghan, S. J., Zhang, Y., Saylor, R. D., Chapman, E. G., Laulainen, N. S., Abdul-Razzak,

H., Leung, L. R., Bian, X., and Zaveri, R. A. (2004). MIRAGE: model description and evaluation of aerosols and trace gases. J. Geophys. Res.-Atmos. 109, D20210.

Slinn, W. G. N. (1984). Precipitation scavenging. U.S. Department of Energy. Retrieved from https://hero.epa.gov/hero/index.cfm/reference/details/reference_id/70298

Grell, G.A. and Devenyi, D. (2002). A Generalized Approach to Parameterizing Convection Combining Ensemble and Data Assimilation Techniques. Geophys. Res. Lett. 29, 1693.

Ordóñez, C. et al (2012). Bromine and iodine chemistry in a global chemistry-climate model: Description and evaluation of very short-lived oceanic sources. Atmospheric Chemistry and Physics, 12, 1423–1447.

Fernandez, R. P., Salawitch, R. J., Kinnison, D. E., Lamarque, J.-F. F. & Saiz-Lopez, A. (2014). Bromine partitioning in the tropical tropopause layer: Implications for stratospheric injection. Atmospheric Chemistry and Physics, 14, 13391–13410.

Fernandez, R.P., Carmona-Balea, A., Cuevas, C.A., Barrera, J.A., Kinnison, D.E., Lamarque, J.F., Blaszczak-Boxe, C., Kim, K., Choi, W., Hay, T. and Blechschmidt, A.M. (2019). Modeling the sources and chemistry of polar tropospheric halogens (Cl, Br, and I) using the CAM-Chem global chemistry-climate model. Journal of Advances in Modeling Earth Systems, 11(7), 2259-2289.

Bobrowski, N., von Glasow, R., Aiuppa, A., Inguaggiato, S., Louban, I., Ibrahim, O. W., and Platt, U. (2007). Reactive halogen chemistry in volcanic plumes. J. Geophys. Res, 112, D06311.

Gómez Martín, J. C., Mahajan, A. S., Hay, T. D., Prados-Román, C., Ordóñez, C., MacDonald, S. M., Plane, J. M. C., Sorribas, M., Gil, M., Paredes Mora, J. F., Agama Reyes, M. V., Oram, D. E., Leedham, E., and Saiz-Lopez, A. (2013). Iodine chemistry in the eastern Pacific marine boundary layer. J. Geophys. Res.-Atmos., 118, 887–904.

Li, Q., Tham, Y.J., Fernandez, R.P., He, X.C., Cuevas, C.A. and Saiz-Lopez, A. (2022). Role of Iodine Recycling on Sea-Salt Aerosols in the Global Marine Boundary Layer. Geophysical Research Letters, 49(6), e2021GL097567.

Saiz-Lopez, A., Blaszczak-Boxe, C. S. and Carpenter, L. J. (2015). A mechanism for biologically induced iodine emissions from sea ice. Atmos. Chem. Phys., 15, 9731–9746.

Saiz-Lopez, A., Fernandez, R.P., Li, Q., Cuevas, C. A., Fu, X., Kinnison, D. E., Tilmes, S., Mahajan, A. S., Gómez Martín, J. C., Iglesias-Suarez, F., Hossaini, R., Plane, J. M. C., Myhre, G. and Lamarque, J. (2023). Natural short-lived halogens exert an indirect cooling effect on climate, Nature, 618, 967–973.

Barrera, J. A., Kinnison, D. E., Fernandez, R. P., Lamarque, J.-F., Cuevas, C. A., Tilmes, S. and Saiz-Lopez, A. (2023). Comparing the effect of anthropogenically amplified halogen natural emissions on tropospheric ozone chemistry between pre-industrial and present-day. J. Geophys. Res.-Atmos., 128, e2022JD038283.

Yuan, Y., Zhang, Y., Mao, J., Yu, G., Xu, K., Zhao, J., Qian, H., Wu, L., Yang, X., Chen, Y. and Ma, W. (2023). Diverse changes in shipping emissions around the Western Pacific ports under the coeffect of the epidemic and fuel oil policy. Science of The Total Environment, 879, 162892.

Fan, Q., Zhang, Y., Ma, W., Ma, H., Feng, J., Yu, Q., et al. (2016). Spatial and Seasonal Dynamics of Ship Emissions over the Yangtze River Delta and East China Sea and Their Potential Environmental Influence. Environmental Science & Technology, 50(3), 1322–1329.

Li, S., Sarwar, G., Zhao, J., Zhang, Y., Zhou, S., Chen, Y., et al. (2020). Modeling the impact of marine DMS emissions on summertime air quality over the coastal East China seas. Earth and Space Science, 7, e2020EA001220.

Veres, P. R., Neuman, J. A., Bertram, T. H., Assaf, E., Wolfe, G. M., Williamson, C. J., et al. (2020). Global airborne sampling reveals a previously unobserved dimethyl sulfide oxidation mechanism in the marine atmosphere, Proceedings of the National Academy of Sciences, 117(9), 4505-4510.

Thalman, R. and Volkamer, R. (2013). Temperature dependent absorption cross-sections of O2-O2 collision pairs between 340 and 630 nm and at atmospherically relevant pressure. Physical Chemistry Chemical Physics, 15, 15371-15381.

Serdyuchenko, A., Gorshelev, V., Weber, M., Chehade, W., and Burrows, J.P. (2014). High spectral resolution ozone absorption cross-sections-part 2: temperature dependence, Atmospheric Measurement Techniques, 7, 625-636.

Wilmouth, D.M., Hanisco, T.F., Donahue, M.D., and Anderson, J.G. (1999). Fourier Transform Ultraviolet Spectroscopy of the A Pi-2(3/2) Direct Current X Pi-2(3/2) Transition of BrO. Journal of Physical Chemistry A, 103, 8935-8945.

Vandaele, A.C., Hermans, C., Simon, P.C., Carleer, M., Colin, R., Fally, S., et al. (1998). Measurements of the NO_2 absorption crosssection from 42000 cm-1 to 10000 cm−1 (238-1000 nm) at 220 K and 294 K. Journal of Quantitative Spectroscopy and Radiative Transfer, 59, 171-184.

Rothman, L.S., Gordon, I.E., Barber, R.J., Dothe, H., Gamache, R.R., Goldman, A., et al. (2010). HITEMP, the high-temperature molecular spectroscopic database. Journal of Quantitative Spectroscopy and Radiative Transfer, 111, 2139-2150.

Iacono, M.J., Delamere, J.S., Mlawer, E.J., Shephard, M.W., Clough, S.A. and Collins, W.D. (2008). Radiative forcing by long-lived greenhouse gases: Calculations with the AER radiative transfer models. Journal of Geophysical Research: Atmospheres, 113, 2–9.

Chen, F. and Dudhia, J. (2001). Coupling an Advanced Land Surface–Hydrology Model with the Penn State–NCAR MM5 Modeling System. Part II: Preliminary Model Validation. Monthly Weather Review, 129, 587–604, 2001.

Morrison, H., Thompson, G. and Tatarskii, V. (2008). Impact of Cloud Microphysics on the Development of Trailing Stratiform Precipitation in a Simulated Squall Line: Comparison of One- and Two-Moment Schemes. Monthly Weather Review, 137, 991–1007.

Sukoriansky, S., Galperin, B. and Perov, V. (2005). Application of a new spectral theory of stably stratified turbulence to the atmospheric boundary layer over sea ice. Boundary-Layer Meteorology, 117, 231–257.

Emmons, L. K., Walters, S., Hess, P. G., Lamarque, J.-F. F., Pfister, G. G., Fillmore, D., Granier, C., Guenther, A., Kinnison, D., Laepple, T., Orlando, J., Tie, X., Tyndall, G., Wiedinmyer, C., Baughcum, S. L. and Kloster, S. (2010). Description and evaluation of the Model for Ozone and Related chemical Tracers, version 4 (MOZART-4). Geoscientific Model Development, 3(1), 43–67.

Zaveri, R. A., Easter, R. C., Fast, J. D. and Peters, L. K. (2008). Model for Simulating Aerosol Interactions and Chemistry (MOSAIC). Journal of Geophysical Research: Atmospheres, 113(13), 1–29.

Tie, X. (2003). Effect of clouds on photolysis and oxidants in the troposphere. Journal of Geophysical Research, 108, 4642.

Archer-Nicholls, S., Lowe, D., Utembe, S., Allan, J., Zaveri, R. A., Fast, J. D., Hodnebrog, Denier Van Der Gon, H. and McFiggans, G. (2014). Gaseous chemistry and aerosol mechanism developments for version 3.5.1 of the online regional model, WRF-Chem. Geoscientific Model Development, 7(6), 2557–2579.

Guenther, A., Karl, T., Harley, P., Wiedinmyer, C., Palmer, P.I. and Geron, C. (2006). Estimates of global terrestrial isoprene emissions using MEGAN (Model of Emissions of Gases and Aerosols from Nature). Atmospheric Chemistry and Physics, 6(11), 3181-3210.

Wiedinmyer, C., Akagi, S. K., Yokelson, R. J., Emmons, L. K., Al-Saadi, J. A., Orlando, J. J. and Soja, A. J. (2011). The Fire INventory from NCAR (FINN): A high resolution global model to estimate the emissions from open burning. Geoscientific Model Development, 4(3), 625–641.

Wang, Y. Q. (2014). MeteoInfo: GIS software for meteorological data visualization and analysis. Meteorological Applications, 21(2), 360-368.
